# Supplementary material for: Assessment of heavy metal pollution in sediments from the urban section of Yihe River, Linyi City, China
Source: PLoS One. 2025 Feb 13;20(2):e0318579. doi: 10.1371/journal.pone.0318579 (PMC11824964; doi:10.1371/journal.pone.0318579)
Supplement: S1 Table — (DOCX) [file pone.0318579.s001.docx]

**S1 Table. The distribution of specific metals.**

| Sampling points | Cr | Ni | Cu | Zn | Cd | Pb | As | Hg |
| --- | --- | --- | --- | --- | --- | --- | --- | --- |
| DN01 | 67.63 | 36.30 | 37.50 | 169.21 | 0.45 | 45.28 | 7.39 | 0.44 |
| DN02 | 59.70 | 37.52 | 29.34 | 165.66 | 0.18 | 28.13 | 5.73 | 0.05 |
| DN03 | 33.03 | 36.09 | 14.97 | 87.32 | 0.14 | 24.25 | 2.35 | 0.07 |
| DN04 | 31.63 | 7.16 | 3.17 | 23.19 | 0.03 | 17.64 | 1.00 | 0.01 |
| DN05 | 74.97 | 40.18 | 29.24 | 115.46 | 0.16 | 28.09 | 7.93 | 0.07 |
| DN06 | 34.08 | 18.59 | 6.89 | 68.26 | 0.08 | 18.32 | 1.77 | 0.02 |
| DN07 | 26.98 | 12.47 | 4.88 | 39.20 | 0.04 | 18.84 | 1.34 | 0.02 |
| DN08 | 34.73 | 14.67 | 5.73 | 51.04 | 0.03 | 18.68 | 1.41 | 0.01 |
| DN09 | 46.89 | 15.45 | 6.59 | 48.74 | 0.08 | 18.09 | 1.50 | 0.01 |
| DN10 | 25.35 | 14.32 | 10.76 | 129.25 | 0.61 | 29.14 | 1.62 | 0.02 |
| DN11 | 12.83 | 6.35 | 2.29 | 15.48 | 0.03 | 16.15 | 1.24 | 0.01 |
| DN12 | 23.26 | 6.54 | 3.51 | 21.28 | 0.09 | 17.19 | 1.34 | 0.01 |
| DN13 | 24.09 | 5.85 | 2.59 | 15.51 | 0.04 | 17.83 | 0.93 | 0.01 |
| DN14 | 34.30 | 13.29 | 6.17 | 43.49 | 0.06 | 18.69 | 1.52 | 0.01 |
| DN15 | 35.30 | 14.06 | 6.76 | 39.66 | 0.13 | 20.33 | 1.11 | 0.01 |
| DN16 | 25.39 | 11.73 | 4.64 | 31.01 | 0.03 | 15.18 | 1.53 | 0.01 |
| DN17 | 42.25 | 13.61 | 5.39 | 41.41 | 0.04 | 16.67 | 1.73 | 0.05 |
| DN18 | 41.21 | 14.68 | 9.17 | 53.12 | 0.08 | 17.78 | 1.92 | 0.01 |
| DN19 | 20.16 | 4.51 | 3.03 | 12.32 | 0.03 | 16.18 | 1.35 | 0.01 |
| DN20 | 30.78 | 11.06 | 5.70 | 28.62 | 0.04 | 18.43 | 1.39 | 0.01 |
| DN21 | 30.26 | 10.43 | 5.68 | 31.32 | 0.05 | 15.30 | 1.28 | 0.01 |
| DN22 | 34.05 | 10.23 | 5.83 | 30.93 | 0.09 | 18.65 | 1.24 | 0.01 |
| DN23 | 60.55 | 35.14 | 27.90 | 173.31 | 0.25 | 28.31 | 6.07 | 0.09 |
| DN24 | 29.90 | 6.59 | 5.35 | 14.86 | 0.09 | 18.07 | 0.80 | 0.01 |
| DN25 | 38.65 | 8.73 | 4.22 | 18.56 | 0.20 | 18.64 | 0.90 | 0.03 |
