# Supplementary material for: Assessment of heavy metal pollution in sediments from the urban section of Yihe River, Linyi City, China
Source: PLoS One. 2025 Feb 13;20(2):e0318579. doi: 10.1371/journal.pone.0318579 (PMC11824964; doi:10.1371/journal.pone.0318579)
Supplement: S2 Table — (DOCX) [file pone.0318579.s002.docx]

**S2 Table. *PLI* values calculated at each sampling point.**

| Sampling points | *PLI* |
| --- | --- |
| DN01 | 2.49375 |
| DN02 | 1.46704 |
| DN03 | 1.0257 |
| DN04 | 0.31667 |
| DN05 | 1.55755 |
| DN06 | 0.60395 |
| DN07 | 0.45548 |
| DN08 | 0.46967 |
| DN09 | 0.55398 |
| DN10 | 0.89279 |
| DN11 | 0.23899 |
| DN12 | 0.36066 |
| DN13 | 0.27488 |
| DN14 | 0.4948 |
| DN15 | 0.52322 |
| DN16 | 0.3824 |
| DN17 | 0.56586 |
| DN18 | 0.60416 |
| DN19 | 0.25727 |
| DN20 | 0.39968 |
| DN21 | 0.40519 |
| DN22 | 0.46989 |
| DN23 | 1.66589 |
| DN24 | 0.33428 |
| DN25 | 0.50696 |
