# Supplementary material for: Assessment of heavy metal pollution in sediments from the urban section of Yihe River, Linyi City, China
Source: PLoS One. 2025 Feb 13;20(2):e0318579. doi: 10.1371/journal.pone.0318579 (PMC11824964; doi:10.1371/journal.pone.0318579)
Supplement: S3 Table — (DOCX) [file pone.0318579.s003.docx]

**S3 Table. *I_geo_* values calculated at each sampling point.**

| Sampling points | *I_geo_*(Cr) | *I_geo_*(Ni) | *I_geo_*(Cu) | *I_geo_*(Zn) | *I_geo_*(Cd) | *I_geo_*(Pb) | *I_geo_*(As) | *I_geo_*(Hg) |
| --- | --- | --- | --- | --- | --- | --- | --- | --- |
| DN01 | -0.32 | 0.04 | 0.35 | 1.01 | 1.44 | 0.25 | -0.52 | 3.62 |
| DN02 | -0.50 | 0.09 | 0.00 | 0.98 | 0.11 | -0.44 | -0.89 | 0.39 |
| DN03 | -1.35 | 0.03 | -0.97 | 0.05 | -0.27 | -0.65 | -2.17 | 0.95 |
| DN04 | -1.41 | -2.30 | -3.21 | -1.86 | -2.43 | -1.11 | -3.40 | -2.23 |
| DN05 | -0.17 | 0.19 | -0.01 | 0.46 | -0.06 | -0.44 | -0.42 | 0.88 |
| DN06 | -1.31 | -0.92 | -2.09 | -0.30 | -0.98 | -1.06 | -2.58 | -1.26 |
| DN07 | -1.64 | -1.50 | -2.59 | -1.10 | -2.12 | -1.02 | -2.98 | -0.80 |
| DN08 | -1.28 | -1.26 | -2.36 | -0.72 | -2.25 | -1.03 | -2.90 | -1.59 |
| DN09 | -0.85 | -1.19 | -2.16 | -0.79 | -1.00 | -1.08 | -2.82 | -1.63 |
| DN10 | -1.73 | -1.30 | -1.45 | 0.62 | 1.89 | -0.39 | -2.71 | -0.92 |
| DN11 | -2.72 | -2.47 | -3.68 | -2.44 | -2.72 | -1.24 | -3.09 | -2.84 |
| DN12 | -1.86 | -2.43 | -3.07 | -1.98 | -0.84 | -1.15 | -2.99 | -2.14 |
| DN13 | -1.81 | -2.59 | -3.50 | -2.44 | -2.09 | -1.10 | -3.50 | -2.56 |
| DN14 | -1.30 | -1.41 | -2.25 | -0.95 | -1.42 | -1.03 | -2.80 | -1.64 |
| DN15 | -1.26 | -1.33 | -2.12 | -1.09 | -0.31 | -0.91 | -3.25 | -1.90 |
| DN16 | -1.73 | -1.59 | -2.66 | -1.44 | -2.38 | -1.33 | -2.79 | -1.85 |
| DN17 | -1.00 | -1.37 | -2.45 | -1.02 | -1.97 | -1.19 | -2.62 | 0.37 |
| DN18 | -1.03 | -1.26 | -1.68 | -0.66 | -1.00 | -1.10 | -2.47 | -1.29 |
| DN19 | -2.06 | -2.97 | -3.28 | -2.77 | -2.44 | -1.24 | -2.97 | -2.63 |
| DN20 | -1.45 | -1.67 | -2.37 | -1.56 | -2.14 | -1.05 | -2.92 | -2.10 |
| DN21 | -1.48 | -1.76 | -2.37 | -1.43 | -1.64 | -1.32 | -3.04 | -2.07 |
| DN22 | -1.31 | -1.79 | -2.33 | -1.44 | -0.84 | -1.03 | -3.09 | -1.57 |
| DN23 | -0.48 | 0.00 | -0.08 | 1.04 | 0.59 | -0.43 | -0.80 | 1.37 |
| DN24 | -1.50 | -2.42 | -2.46 | -2.50 | -0.82 | -1.08 | -3.72 | -2.84 |
| DN25 | -1.12 | -2.01 | -2.80 | -2.18 | 0.31 | -1.03 | -3.56 | -0.11 |
