# Supplementary material for: Assessment of heavy metal pollution in sediments from the urban section of Yihe River, Linyi City, China
Source: PLoS One. 2025 Feb 13;20(2):e0318579. doi: 10.1371/journal.pone.0318579 (PMC11824964; doi:10.1371/journal.pone.0318579)
Supplement: S4 Table — (DOCX) [file pone.0318579.s004.docx]

**S4 Table. *RI* values calculated at each sampling point.**

| Sampling points | E(Cr) | E(Ni) | E(Cu) | E(Zn) | E(Cd) | E(Pb) | E(As) | E(Hg) | *RI* |
| --- | --- | --- | --- | --- | --- | --- | --- | --- | --- |
| DN01 | 2.41 | 7.72 | 9.57 | 3.02 | 121.78 | 8.91 | 10.48 | 735.94 | 899.82 |
| DN02 | 2.12 | 7.98 | 7.49 | 2.95 | 48.42 | 5.54 | 8.12 | 78.87 | 161.49 |
| DN03 | 1.18 | 7.68 | 3.82 | 1.56 | 37.25 | 4.77 | 3.33 | 115.67 | 175.25 |
| DN04 | 1.13 | 1.52 | 0.81 | 0.41 | 8.35 | 3.47 | 1.42 | 12.83 | 29.95 |
| DN05 | 2.67 | 8.55 | 7.46 | 2.06 | 43.12 | 5.53 | 11.25 | 110.69 | 191.32 |
| DN06 | 1.21 | 3.95 | 1.76 | 1.22 | 22.89 | 3.61 | 2.51 | 25.00 | 62.14 |
| DN07 | 0.96 | 2.65 | 1.25 | 0.70 | 10.39 | 3.71 | 1.89 | 34.38 | 55.92 |
| DN08 | 1.24 | 3.12 | 1.46 | 0.91 | 9.45 | 3.67 | 2.00 | 19.90 | 41.76 |
| DN09 | 1.67 | 3.29 | 1.68 | 0.87 | 22.55 | 3.56 | 2.13 | 19.41 | 55.16 |
| DN10 | 0.90 | 3.05 | 2.74 | 2.30 | 166.79 | 5.73 | 2.30 | 31.74 | 215.56 |
| DN11 | 0.46 | 1.35 | 0.58 | 0.28 | 6.85 | 3.18 | 1.76 | 8.39 | 22.84 |
| DN12 | 0.83 | 1.39 | 0.90 | 0.38 | 25.09 | 3.38 | 1.89 | 13.65 | 47.51 |
| DN13 | 0.86 | 1.24 | 0.66 | 0.28 | 10.57 | 3.51 | 1.33 | 10.20 | 28.64 |
| DN14 | 1.22 | 2.83 | 1.57 | 0.78 | 16.80 | 3.68 | 2.15 | 19.24 | 48.27 |
| DN15 | 1.26 | 2.99 | 1.72 | 0.71 | 36.30 | 4.00 | 1.57 | 16.12 | 64.67 |
| DN16 | 0.90 | 2.50 | 1.18 | 0.55 | 8.65 | 2.99 | 2.17 | 16.61 | 35.55 |
| DN17 | 1.50 | 2.90 | 1.38 | 0.74 | 11.49 | 3.28 | 2.45 | 77.30 | 101.04 |
| DN18 | 1.47 | 3.12 | 2.34 | 0.95 | 22.54 | 3.50 | 2.72 | 24.51 | 61.14 |
| DN19 | 0.72 | 0.96 | 0.77 | 0.22 | 8.32 | 3.18 | 1.91 | 9.70 | 25.79 |
| DN20 | 1.10 | 2.35 | 1.45 | 0.51 | 10.19 | 3.63 | 1.98 | 13.98 | 35.19 |
| DN21 | 1.08 | 2.22 | 1.45 | 0.56 | 14.40 | 3.01 | 1.82 | 14.31 | 38.84 |
| DN22 | 1.21 | 2.18 | 1.49 | 0.55 | 25.21 | 3.67 | 1.76 | 20.23 | 56.30 |
| DN23 | 2.15 | 7.48 | 7.12 | 3.09 | 67.53 | 5.57 | 8.61 | 155.10 | 256.65 |
| DN24 | 1.06 | 1.40 | 1.36 | 0.26 | 25.51 | 3.56 | 1.14 | 8.39 | 42.69 |
| DN25 | 1.38 | 1.86 | 1.08 | 0.33 | 55.71 | 3.67 | 1.27 | 55.43 | 120.72 |
