# Supplementary material for: Assessment of heavy metal pollution in sediments from the urban section of Yihe River, Linyi City, China
Source: PLoS One. 2025 Feb 13;20(2):e0318579. doi: 10.1371/journal.pone.0318579 (PMC11824964; doi:10.1371/journal.pone.0318579)
Supplement: S5 Table — (DOCX) [file pone.0318579.s005.docx]

**S5 Table. *TRI* values calculated at each sampling point.**

| Sampling points | *TRI_i_* | | | | | | | | *TRI* |
| --- | --- | --- | --- | --- | --- | --- | --- | --- | --- |
| DN01 | 1.39 | 1.59 | 0.75 | 1.04 | 0.53 | 0.98 | 0.94 | 1.95 | 7.23 |
| DN02 | 1.23 | 1.65 | 0.59 | 1.02 | 0.21 | 0.61 | 0.73 | 0.21 | 6.03 |
| DN03 | 0.68 | 1.59 | 0.30 | 0.54 | 0.16 | 0.52 | 0.30 | 0.31 | 4.09 |
| DN04 | 0.65 | 0.31 | 0.06 | 0.14 | 0.04 | 0.38 | 0.13 | 0.03 | 1.72 |
| DN05 | 1.54 | 1.76 | 0.59 | 0.71 | 0.19 | 0.61 | 1.01 | 0.29 | 6.41 |
| DN06 | 0.70 | 0.82 | 0.14 | 0.42 | 0.10 | 0.40 | 0.22 | 0.07 | 2.80 |
| DN07 | 0.55 | 0.55 | 0.10 | 0.24 | 0.05 | 0.41 | 0.17 | 0.09 | 2.06 |
| DN08 | 0.71 | 0.64 | 0.12 | 0.32 | 0.04 | 0.40 | 0.18 | 0.05 | 2.41 |
| DN09 | 0.96 | 0.68 | 0.13 | 0.30 | 0.10 | 0.39 | 0.19 | 0.05 | 2.76 |
| DN10 | 0.52 | 0.63 | 0.22 | 0.80 | 0.73 | 0.63 | 0.21 | 0.08 | 3.73 |
| DN11 | 0.26 | 0.28 | 0.05 | 0.10 | 0.03 | 0.35 | 0.16 | 0.02 | 1.22 |
| DN12 | 0.48 | 0.29 | 0.07 | 0.13 | 0.11 | 0.37 | 0.17 | 0.04 | 1.62 |
| DN13 | 0.49 | 0.26 | 0.05 | 0.10 | 0.05 | 0.39 | 0.12 | 0.03 | 1.45 |
| DN14 | 0.70 | 0.58 | 0.12 | 0.27 | 0.07 | 0.40 | 0.19 | 0.05 | 2.35 |
| DN15 | 0.72 | 0.62 | 0.14 | 0.24 | 0.16 | 0.44 | 0.14 | 0.04 | 2.46 |
| DN16 | 0.52 | 0.52 | 0.09 | 0.19 | 0.04 | 0.33 | 0.19 | 0.04 | 1.88 |
| DN17 | 0.87 | 0.60 | 0.11 | 0.26 | 0.05 | 0.36 | 0.22 | 0.20 | 2.46 |
| DN18 | 0.85 | 0.64 | 0.18 | 0.33 | 0.10 | 0.38 | 0.24 | 0.06 | 2.73 |
| DN19 | 0.41 | 0.20 | 0.06 | 0.08 | 0.04 | 0.35 | 0.17 | 0.03 | 1.31 |
| DN20 | 0.63 | 0.49 | 0.11 | 0.18 | 0.04 | 0.40 | 0.18 | 0.04 | 2.03 |
| DN21 | 0.62 | 0.46 | 0.11 | 0.19 | 0.06 | 0.33 | 0.16 | 0.04 | 1.94 |
| DN22 | 0.70 | 0.45 | 0.12 | 0.19 | 0.11 | 0.40 | 0.16 | 0.05 | 2.13 |
| DN23 | 1.24 | 1.54 | 0.56 | 1.07 | 0.30 | 0.61 | 0.77 | 0.41 | 6.10 |
| DN24 | 0.61 | 0.29 | 0.11 | 0.09 | 0.11 | 0.39 | 0.10 | 0.02 | 1.71 |
| DN25 | 0.79 | 0.38 | 0.08 | 0.11 | 0.24 | 0.40 | 0.11 | 0.15 | 2.14 |
