# Supplementary material for: Assessment of heavy metal pollution in sediments from the urban section of Yihe River, Linyi City, China
Source: PLoS One. 2025 Feb 13;20(2):e0318579. doi: 10.1371/journal.pone.0318579 (PMC11824964; doi:10.1371/journal.pone.0318579)
Supplement: S6 Table — (DOCX) [file pone.0318579.s006.docx]

**S6 Table. Characteristic values and cumulative contribution rates of metals in sediments.**

| Total ingredients explained | | | | | | | | | | | |
| --- | --- | --- | --- | --- | --- | --- | --- | --- | --- | --- | --- |
| Element | Initial eigenvalue |  |  | Extract the sum of squared loads |  |  | Rotating load sum of squares |  |  | Rotated component matrix | |
|  | Total | Contribution rate | Grand total% | total | Contribution rate | Grand total% | Total | Contribution rate | Grand total% | PC1 | PC2 |
| Cr | 6.271 | 78.388 | 78.388 | 6.271 | 78.388 | 78.388 | 4.338 | 54.224 | 54.224 | 0.913 | 0.215 |
| Ni | 0.896 | 11.196 | 89.584 | 0.896 | 11.196 | 89.584 | 2.829 | 35.36 | 89.584 | 0.901 | 0.307 |
| Cu | 0.481 | 6.013 | 95.598 |  |  |  |  |  |  | 0.864 | 0.489 |
| Zn | 0.181 | 2.268 | 97.865 |  |  |  |  |  |  | 0.73 | 0.593 |
| Cd | 0.09 | 1.12 | 98.985 |  |  |  |  |  |  | 0.15 | 0.936 |
| Pb | 0.049 | 0.617 | 99.602 |  |  |  |  |  |  | 0.587 | 0.794 |
| As | 0.023 | 0.282 | 99.884 |  |  |  |  |  |  | 0.906 | 0.354 |
| Hg | 0.009 | 0.116 | 100 |  |  |  |  |  |  | 0.475 | 0.682 |
