# Supplementary material for: Assessment of heavy metal pollution in sediments from the urban section of Yihe River, Linyi City, China
Source: PLoS One. 2025 Feb 13;20(2):e0318579. doi: 10.1371/journal.pone.0318579 (PMC11824964; doi:10.1371/journal.pone.0318579)
Supplement: Attachment — Submitted filename: Manuscript PLOS ONE AE.docx [file pone.0318579.s010.docx]

Assessment of heavy metal pollution in sediments from the urban section of Yihe River, Linyi City, China

**Qinghai Deng^1^, Guizong Sun^1^, Fuquan Li^2^, Lei Guo^2^ , Dan Shi^2^, Liping Zhang^1^**^*^**, Zhenzhou Sun^1^, Jingjing Yang^1^**

^1^ Collage of Earth Sciences and Engineering, Shandong University of Science and Technology, Qingdao, Shandong Province, China

^2^ The Seventh Geological Brigade of Shandong Provincial Bureau of Geology and Mineral Resources, Linyi, Shandong Province, China

*[zhangliping@sdust.edu.cn](mailto:zhangliping@sdust.edu.cn) (LZ)

**Abstract**: The Yihe River is the largest mountain torrent river in Shandong Province, which plays an important role in flood control, irrigation and climate regulation. Due to the impacts of the upstream and urban domestic sewage, as well as industrial and agricultural wastewater, the Linyi City section of the Yihe River is expected to have a high risk of heavy metal (HM) pollution. Sediments are the main reservoir and potential release source of heavy metals in river systems. Assessment of heavy metals in sediments can identify anthropogenic pollution. In this study, 25 sediment samples were collected from the Linyi City section of the Yihe River and its tributaries, and the concentration of eight HMs (Cr, Cu, Pb, Zn, Ni, Cd, As, Hg) weredetermined. The pollution levels were evaluated by determining the pollution load index, geoaccumulation index, etc. Correlation analysis and absolute principal component-multiple linear regression (APCS-MLR) were used to conduct source apportionment. Cr, Cu, Pb, Zn, Ni, Cd, As, and Hg were detected in all sediment samples. Overall, the concentration of HMs in the sediments of the main stream of the Yihe River is mostly within the environmental background value, and the overall state is from no pollution to slightly polluted, while the tributaries of the Yihe River are in a slightly polluted state. Hg and Cd are the two main HMs pollutants in the surface sediments of the study area, with the average content of 1.65 and 1.11 times the background value, respectively. Most areas of the main stream of the Yihe River are free of HM pollution, with low ecological risk and no toxicity risk. The ecological risks in the tributaries (Suhe River, Benghe River, Liuqinghe River) and the river center island (Yihe River North Island) are high and assessed as presenting low toxicity. HMs have no non-carcinogenic risk to humans. Cr is the main carcinogenic factor and has an unacceptable carcinogenic risk to children. Source analysis showed that Cr, Ni, Cu, Zn, and As mainly come from natural sources and agricultural activities, while Cd, Pb, and Hg are mainly the result of transportation and industry. The results help us understand the distribution and pollution of HMs in the river sediments, and also provide management support to local environmental management departments and relevant national departments.

**Keywords**: Heavy metals; Surface sediments; Pollution assessment; Source apportionment

# Introduction

Heavy metals (HMs) come from a wide range of sources, are highly toxic, persistent and non-biodegradable, thus have attracted increasing attention from researchers [1-3]. In recent decades, the exponential growth of the global population and economy, as well as the development of industry, agriculture and urbanization, have jointly led to the introduction of large amounts of HMs into rivers, resulting in the decline of water quality, and increased HM pollution and ecological risks [4-6]. HMs can be enriched in sediments by physical adsorption, chemical precipitation and aquatic degradation. Sediments can store and accumulate heavy metals from industrial emissions, agricultural activities, urban sewage discharge, and atmospheric deposition for a long time, making the heavy metal content in water bodies lower. When disturbed or hydrodynamic conditions change, HMs in sediments will re-enter the water body as the sediment-water interface environment alters and become a source of secondary pollution [7-9]. HMs can also have a negative impact on aquatic ecosystems through bioaccumulation and biomagnification processes. If local residents continue to be exposed to heavy metals exceeding the safe limit, it may have harmful effects and lead to non-carcinogenic risks such as neurological diseases, liver and kidney diseases [9-12]. Children in particular, whose physical development is not yet fully mature, have relatively weak functions of their organs and systems, and have limited ability to metabolize and excrete heavy metals. Sediments in river systems are more representative of their pollution status [13] and have become valuable environmental archives for identifying anthropogenic pollution and metal enrichment in river environments [14]. The assessment of sediment quality can provide information on pollution and environmental conditions in aquatic ecosystems [15]. Therefore, studies on the characteristics, source factors and ecological effects of HM pollution in sediments are crucial to better control HM pollution in the environment [16].

Yihe River, a major tributary of the Yishu-Si River system within the Huaihe River Basin, stretches across the southern part of Shandong Province and the northern part of Jiangsu Province in southeast China, with a total length of 574 kilometers. Within this system, the Linyi section spans 226 kilometers and flows through Yishui County, Yinan County, urban areas, Lanling County, and Tancheng County in Linyi City. As the largest river in Linyi City, the Yihe River has a complete ecosystem structure and function, rich biodiversity, and along with its tributaries, it plays an important role in flood control, irrigation, climate regulation, and maintaining ecological balance. However, the urban section of the Yihe River and its tributaries in Linyi City are seriously affected by domestic sewage, industrial wastewater and agricultural non-point source pollution from upstream counties and three districts under the jurisdiction of Linyi City [17], which may become potential sources of HMs in the sediments of the Yihe River. Previous studies mainly analyzed the characteristics of HMs pollution in the sediments of the Yihe River [18-20], but did not address the source of HMs pollutants in the sediments of the urban section of the Yihe River and its tributaries in Linyi City. Therefore, the objectives of this study were: (1) to describe the distribution characteristics of eight HMs (Cd, As, Pb, Zn, Cu, Hg, Ni, Cr, Cd) in sediments; (2) to evaluate the pollution status, ecological risks and risks to human health of heavy metals; and (3) to determine the possible sources of HMs through APCS-MLR and Pearson correlation analysis. The results are expected to enhance our understanding of HM pollution in the Yihe River and provide a reference for pollution control and ecological restoration by relevant local departments.

# Materials and Methods

## Overview of the study area

The Yihe River, located in eastern China, originates from the northern foot of Niujiao Mountain in Yiyuan County, Zibo City, Shandong Province. It flows through Yishui County, Yinan County, Mengyin County, Pingyi County, as well as other counties in Linyi City, and into the Yellow Sea from Jiangsu Province. It has a total length of 574 km and a drainage area of 17325 km^2^. The Yihe River Basin has a temperate continental monsoon climate with four distinct seasons, and rain and heat occurring at the same time. The annual average temperature is 13.4~15℃, and the average annual rainfall is 800~848 mm. Three tributaries, including the Benghe River, Liuqinghe River, and Suhe River, merge into the Yihe River in the northeast of Linyi City [21]. In the 1990s, Linyi City built the longest Xiaobudong Rubber Dam in Asia on the main stream of the Yihe River for water storage, forming a vast water surface of 1066.67 hm^2^ (also known as Yimeng Lake) in Linyi City [20]. Due to the rapid development of industry and agriculture in Linyi in recent decades and the accelerated increase in the population, the Yihe River and its tributaries may be contaminated by HMs [17, 18, 21].

## Sample collection and analysis

In February 2023, a total of 25 sediment samples were collected in the Yihe River Basin from the 0-20 cm layer (Fig 1). This was performed using a portable sediment sampler, the samples were placed in clean polyethylene bags, sealed and numbered, and stored in a 4℃ freezer. The geographical location was recorded using a GPS device. The sediment was air-dried at room temperature, made into powder, and sieved through a 100-mesh screen to remove impurities such as plant residues and gravel in the sediment. After grinding, it was stored in a polyethylene bag for later use. No special permission was required to take samples from the Yihe River. The study area was not privately owned or protected in any way, and the field research did not involve endangered or protected species.

**Fig 1. Geographical location and sampling points of the study area**

(Map data comes from Natural Earth. <http://www.naturalearthdata.com/>)

All analyses were performed at the laboratory of The Seventh Geological Brigade of Shandong Provincial Bureau of Geology and Mineral Resources. According to the method of "Solid Waste - Metal Inductively Coupled Plasma Mass Spectrometry Determination" (HJ766-2015), the concentrations of As, Cd, Cr, Cu, Ni, Pb and Zn in the sediments of the Yihe River were quantified by inductively coupled plasma mass spectrometry (ICP-MS). Briefly, 0.10 g of sediment powder was taken, HCl (1.0 mL), HNO_3_ (4.0 mL), HF (1.0 mL), and H_2_O_2_ (1.0 mL) were added, the internal solutes were dissolved and transferred to a volumetric bottle, and deionized water was added for the volume to reach 50 mL as the sample. The concentration of HMs was measured using an inductively coupled plasma mass spectrometer (iCAP RQ, USA). According to the "Soil and Sediment - Determination of Mercury, Arsenic, Selenium, Bismuth, Antimony - Microwave Dissolution/Atomic Fluorescence Method" (HJ680-2013), the concentration of Hg in soil samples was determined by atomic fluorescence spectrometry (AFS). To 0.50 g of sediment sample, aqua regia (5.0 mL) and distilled water (5.0 mL) were added, and after digestion, it was transferred to a volumetric flask and diluted to 50 mL by adding deionized water as the sample. The Hg concentration was measured using an atomic fluorescence spectrometer (AFS-8230, China).

To ensure high accuracy, strict quality management and control were adopted for all experimental procedures. A certified reference sediment (GBW07401) was used to determine the analytical accuracy, with relative standard deviations (RSD) of <5% for all samples. The recovery rates for all HMs ranged from 95% to 105%. In addition, the limits of detection (LOD) of each HM were different (Table 1).

**Table 1. Detection limits of HMs (mg/kg)**

|  | Cr | As | Cu | Hg | Ni | Pb | Zn | Cd |
| --- | --- | --- | --- | --- | --- | --- | --- | --- |
| LOD | 1.5 | 0.2 | 0.1 | 0.0005 | 0.2 | 0.2 | 1 | 0.02 |

However, it should be noted that due to research design and resource limitations, no water samples were collected for testing in this study. Therefore, we were unable to directly analyze the relationship and differences between heavy metals in water and sediments. Nevertheless, through detailed sediment analysis, we still revealed some important characteristics of heavy metal pollution in the area.

## Pollution assessment methods

### Contamination Factor (*CF*) and Pollution Load Index (*PLI*)

The pollution situation of a harmful substance can be evaluated by calculating the contamination factor (*CF*) [22]. The calculation formula is as follows:

 (1)

where $C_{metal}$ refers to the actual measured heavy metal concentration in the sediment at a specific location; $C_{background}$ is the heavy metal background value. Based on the *CF*, HM pollution can be divided into four groups: *CF* < 1: low pollution; 1 < *CF* < 3: moderate pollution; 3 < *CF* < 6: considerable pollution; *CF* > 6: high pollution [23].

The Pollution Load Index (*PLI*) is a comprehensive measure that represents information on different elements within an area. *PLI* can reflect spatiotemporal changes and the contribution of each element to total pollution [24]. It is calculated as follows:

$PLI=(CF_{1}\times CF_{2}\times CF_{3}\times.....\times CF_{n})^{1/n}$ (2)

where n denotes the total number of heavy metal elements detected in the sediment sample. Based on *PLI*, samples can be divided into four categoris: *PLI* < 1, unpolluted; 1 ≤ *PLI* < 2, moderately polluted; 2 ≤ *PLI* < 3, highly polluted; *PLI* > 3, extremely polluted [25].

### Geoaccumulation Index (*I_geo_*)

The *I_geo_* index was proposed by Müller [26]. It comprehensively considers the impact of human activities and natural geological processes on the background value of HMs, and is calculated using the following formula:

$I_{geo}=\log_{2}\frac{C_{n}}{1.5\times B_{n}}$ (3)

where *C_n_* represents the measured concentration of metals in sediments; *B_n_* is the geochemical background concentration [27]. Due to the change in lithology, the factor 1.5 is added to the formula as the background matrix correction factor [28]. Based on the *I_geo_* values, samples can be divided into different categories: *I_geo_* < 0: uncontaminated; 0 < *I_geo_* < 1: uncontaminated to moderately polluted; 1 < *I_geo_* < 2: moderately contaminated; 2 < *I_geo_* < 3: moderately to strongly contaminated; 3 < *I _geo_* < 4: strongly contaminated; 4 < *I_geo_* < 5: strongly to extremely contaminated; *I_geo_* > 5: extremely contaminated [29, 30].

## Risk Assessment Methods

### Potential ecological risk assessment

The potential ecological risk index can better evaluate the potential ecological risk factors of heavy metal pollution by combining ecological and environmental effects with toxicology [31]. The following formula is used [22]:

$E_{r}^{i}=T_{r}^{i}C_{f}^{i}$ (4)

$C_{f}^{i}=C_{n}^{i}/C_{o}^{i}$ (5)

$RI=\sum E_{r}^{i}$ (6)

where $E_{r}^{i}$ represents the potential ecological risk of a single element; *RI* represents the sum of the risk aspects of all HMs in the sediment; $T_{r}^{i}$represents the toxicity response factor; $C_{n}^{i}$ represents the heavy metal concentration; $C_{o}^{i}$ is the reference value in this study. The coefficients of elemental toxicity $T_{r}^{i}$ are: Hg = 40, Cd = 30, As = 10, Cu = Pb = Ni = 5, Cr = 2, Zn = 1. The calculated $E_{r}^{i}$ and *RI* value classification is shown in Table 2 [22, 32].

**Table 2.** **Classification of potential risk index**

| *E _r_ ^i^* | Single factor risk level | *RI* | Potential risk level |
| --- | --- | --- | --- |
| *E _r_ ^i^* < 30 | Low risk | RI < 100 | Low |
| 3 0 ≤ *E _r_ ^i^* < 5 0 | Medium risk | 100 ≤ RI < 150 | Medium |
| 5 0 ≤ *E _r_ ^i^* < 1 0 0 | Strong risk | 150 ≤ RI < 200 | Strong |
| 1 0 0 ≤ *E _r_ ^i^* < 15 0 | Very high risk | 200 ≤ RI < 350 | Very high |

### Toxicity Risk Index (*TRI*)

The *TRI* is based on the *TEL* and *PEL* effects to evaluate the toxicity risk of HMs in sediments [28]. The following formula is used for calculation:

$TRI_{i}=\sqrt{\frac{(C_{i}/TEL)^{2}+(C_{i}/PEL)^{2}}{2}}$ (7)

The comprehensive toxicity risk is calculated using the following formula:

$TRI=\sum_{i=1}^{n} TRI_{i}$ (8)

where $TRI_{i}$ represents the toxicity risk index of a single heavy metal; $C_{i}$ represents the concentration of HMs in sediment samples; n represents the number of HMs; $TRI$ represents the toxicity risk index; $TEL$ represents the threshold effect level; $PEL$ represents the possible impact level. The $TRI$ values are divided into different categories: $TRI$ ≤ 5, no toxicity risk; 5 < $TRI$ ≤ 10, low toxicity risk; 10 < $TRI$ ≤ 15, moderate toxicity risk; 15 < $TRI$ ≤ 20, considerable toxicity risk; $TRI$ > 20, very high toxicity risk.

### Human health risk assessment

Heavy metal pollution can seriously affect human health, and assessing human health risk (*HI*) is an effective model for assessing this risk. In general, human exposure to heavy metals in sediments can lead to non-carcinogenic and carcinogenic risks (*CR*) through three pathways: ingestion, inhalation, and dermal contact [33]. This study only considers dermal contact. The Hazard Index (*HI*) is the sum of the average total risk of non-carcinogens for a given element from both routes of exposure (ingestion and dermal) and is calculated using the following formula [36]:

$HI=\sum HQ_{i}=\frac{ADD_{i}}{RfD_{i}}$ (10)

Where *RfD* is the reference dose (mg/kg/day) of each heavy metal, which is the maximum allowable risk of heavy metals to human health. According to USEPA [34], *HI* < 1 represents no significant non-carcinogenic risk, while *HI* ≥ 1 represents adverse effects on human health. The ADD dermal is calculated as follows (Equation 11)

$ADD_{dermal}=\frac{C\times SA\times AF\times ABS\times ED\times EF}{BW\times AT}\times CF$ （11）

Where *ADD_dermal_* (mg/kg/day) is the average daily dose through absorption through dermal contact; *C* represents the concentration of heavy metals (mg/kg). Other parameters are detailed in Supporting Information S1 Table .

The carcinogenic risk (*CR*) estimates the probability that an individual will develop any type of cancer due to exposure to a carcinogen during his or her lifetime [35]. The carcinogenic risk of a heavy metal to an individual's health is calculated using Equation [34] :

$CR=ADD\times SF$ （12）

$TCR=\sum CR$ （13）

Where *CR* is the carcinogenic risk; *TCR* is the total carcinogenic risk; *SF* (dimensionless) is the carcinogenic slope factor. If the *CR* and *TC*R values are less than 1×10^−6^, they are considered negligible; *CR* and *TCR* values in the range of 10^−6^ to 10^−4^ indicate an acceptable or tolerable carcinogenic risk; *CR* and *TCR* values greater than 1×10^−4^ are considered to be potentially harmful to human health [34].

## Statistical analysis

Correlation analysis and principal component analysis are both basic mathematical analysis methods. The former refers to the analysis of two or more correlated variables to measure the degree of correlation between the two variables. It can illustrate the degree of correlation between the eight HMs and the relationship between their sources. A stronger correlation indicates that the sources are closer. To explain the correlation between HM concentrations in the Yihe River, SPSS 27.0 statistical software was used to calculate the Pearson correlation matrix. The APCS-MLR receptor model was employed to quantify the average contribution rate of pollution sources to HMs, thereby quantitatively describing the contribution of pollution sources to elements. The spatial distribution map of different HMs was created using the inverse distance weighted (IDW) package in ArcGIS 10.8. IDW is a geospatial interpolation technique that predicts the values of variable parameters at locations around a sample site.

# Results

## Distribution of HMs in surface sediments

The analytical results for the 25 sediment samples in the study area are shown in Table 3, and the distribution of specific heavy metals is shown in Table S2. The heavy metal elements Cr, Cu, Pb, Zn, Ni, Cd, As, and Hg were detected in all sediment samples. The pH of the sediments in the study area ranged from 7.83 to 8.42, with an average of 8.26, indicating that the sediments of the Yihe River are alkaline. The concentrations of Cr, Ni, Cu, Zn, Cd, Pb, As, and Hg were 12.83-74.97, 4.51-40.18, 2.29-37.50, 12.32-173.31, 0.025-0.61, 15.18-45.28, 0.80-7.93, and 0.005-0.44 mg/kg, respectively. The average concentration order was Zn (58.73 mg/kg) > Cr (36.72 mg/kg) > Pb (20.79 mg/kg) > Ni (16.22 mg/kg) > Cu (9.89 mg/kg) > As (2.26 mg/kg) > Cd (0.12 mg/kg) > Hg (0.04 mg/kg). The maximum value of Cd was 0.45 mg/kg, which is 1.5 times the Chinese soil environmental quality standard (farmland), indicating that there is certain Cd pollution in the study area. The maximum values of Hg and Ni far exceeded the standards of ERL and TEL, but the average values were within the standard limits, indicating the presence of point source pollution. The contents of the other HMs did not exceed the Chinese soil environmental quality standard (farmland), indicating that heavy metal pollution in the Yihe River is not serious. Taking the environmental background values in Shandong Province as a reference, the concentrations of HMs in some sampling points of sediment in the Yi River Basin exceeded the background values, with the maximum values of Cr, Cu, Pb, Zn, Ni, As, and Hg being 1.33, 1.71, 1.91, 3.09, 1.78, 1.12, and 18.33 times the environmental background values, respectively. The maximum values of Zn and Hg exceeded the standard significantly, with the excess rates of Cr, Cu, Pb, Zn, Ni, Cd, As, and Hg at 16%, 20%, 16%, 28%, 32%, 20%, 8%, and 28% respectively. The average concentrations of Hg, Zn, and Cd were 1.65, 1.05, and 1.11 times the background value, indicating a certain heavy metal pollution in sediments in the study area.

**Table 3. Statistical characteristics and relevant standards of HM mass concentrations in sediments of the Yihe River Basin (mg/kg)**

|  | Cr | Ni | Cu | Zn | Cd | Pb | As | Hg | pH |
| --- | --- | --- | --- | --- | --- | --- | --- | --- | --- |
| Average value | 36.72 | 16.22 | 9.89 | 58.73 | 0.12 | 20.79 | 2.26 | 0.04 | 8.26 |
| Maximum | 74.97 | 40.18 | 37.50 | 173.31 | 0.61 | 45.28 | 7.93 | 0.44 | 8.42 |
| Minimum value | 12.83 | 4.51 | 2.29 | 12.32 | 0.025 | 15.18 | 0.80 | 0.005 | 7.83 |
| Median | 34.05 | 13.29 | 5.73 | 39.66 | 0.08 | 18.43 | 1.41 | 0.01 | 8.29 |
| Standard deviation | 15.00 | 11.21 | 9.89 | 51.08 | 0.14 | 6.63 | 2.08 | 0.09 | 0.13 |
| Coefficient of variation | 0.41 | 0.69 | 1.00 | 0.87 | 1.13 | 0.32 | 0.92 | 2.20 | 0.02 |
| Environmental background value | 56.2 | 23.5 | 19.6 | 56.1 | 0.11 | 25.41 | 7.05 | 0.024 | - |
|  |  |  |  |  |  |  |  |  |  |
| ERL value of American sediment quality standard | 81 | 20.9 | 34 | 150 | 1.2 | 46.7 | 8.2 | 0.15 | - |
| TEL Value of Canadian Sediment Quality Standard | 52.3 | 18 | 18.7 | 124 | 0.7 | 30.2 | 7.24 | 0.13 | - |

The IDW interpolation method was used to study the distribution characteristics of Cr, Ni, Cu, Zn, Cd, Pb, As, and Hg in the Yihe River (Fig 2). The results show that the distribution of HMs in the study area exhibits certain spatial differences, and the distribution patterns of the eight HMs are similar. Overall, the concentrations of As, Cu, Ni, Pb, Ni, Pb, and Zn are low in most areas of the main stream of the Yihe River, while there are two places where the concentrations are significantly higher than other points, namely, sampling points DN05 and DN23. Compared with the main stream of the Yihe River, the eight HMs show higher concentrations in the Yihe River tributaries Suhe (DN01), Benghe (DN02), and Liuqinghe River (DN03).

**Fig 2. Spatial distribution of heavy metal parameters**

## Evaluation of heavy metal pollution

The contamination factor (*CF*) is a clear representation of the distribution of trace elements. The average values of the single factor pollution index of the eight HMs measured in the sediments of the Yihe River, ranked from large to small, were Hg (1.65) > Cd (1.11) > Zn (1.05) > Pb (0.82) > Ni (0.69) > Cr (0.65) > Cu (0.50) > As (0.32). According to the classification standard, Hg, Cd, and Zn are in a medium pollution state, and Pb, Ni, Cr, Cu, and As are in a non-polluted state, indicating that Hg, Cd and Zn are more likely to have adverse ecological effects. The *PLI* of sediments is generally used to estimate the pollution level associated with multiple pollutants in a specific location [36]. According to the displayed *PLI* values (Fig 3a), the pollution levels of different sampling points vary greatly. DN01 is highly polluted (*PLI*=2.49), DN02, DN03, DN05, and DN23 are in a moderately polluted state. The probability index distribution of *PLI* shows that 96% of PLI is below 1.65, which means it is polluted to moderately polluted.

**Fig 3. PLI distribution of heavy metal(a) and probability index distribution of PLI(b)**

Fig 4a presents the box plot of the average *I_geo_* values for 8 HMs. The *I_geo_* values for Cr, Ni, Cu, Zn, Cd, Pb, As, and Hg ranged from -2.72 to 0.17, -2.97 to 0.19, -3.69 to 0.35, -2.77 to 1.04, -2.72 to 1.89, -1.33 to 0.25, -3.72 to 0.42, -2.84 to 3.62, respectively. The values are arranged in descending order of the average values as follows: Pb (-0.93) > Zn (-0.98) > Cd (-1.02) > Hg (-1.06) > Cr (-1.36) > Ni (-1.41) > Cu (-2.06) > As (-2.60). The difference in *I_geo_* shows that the concentration of HMs in the Linyi City section of the Yihe River varies greatly, and there is an obvious point source pollution. The different colors in (Fig 4b) represent the *I_geo_* category of each heavy metal at DN01~DN25. It can be seen that Hg pollution has great spatial variation. The degree of Hg pollution is the highest at DN01, where *I_geo_* reaches 3.62, which is serious pollution, while it shows moderate pollution at DN23. The *I_geo_* of Hg at other sampling points is below 1, indicating no pollution to mild pollution. The pollution of Zn and Cd reaches the maximum at DN01, reaching moderate pollution, and it shows no-pollution ~ mild pollution at other sampling points. The *I_geo_* of Ni, Cu and Pb is between 0 and 1, indicating no pollution to mild pollution; Cr and As show no pollution at all sampling points, and *I_geo_* is negative. Overall, the *I_geo_* values of Cu, Zn, Cd, and Hg at DN01 are higher than those at other sampling points, indicating that heavy metal pollution at DN01 is more serious than that at other sampling points.

**Fig 4.** **(a) *I_geo_* box plot of HMs in the sediments and (b) block diagrams of pollution categories at different sampling points**

## Heavy metal risk assessment

The comprehensive potential ecological risk index (*RI*) and toxicity risk index (*TRI*) of Yihe River were calculated and shown in Fig 5. It can be seen that there are large differences in the *RI* and *TRI* values at various points in the Yihe River, which is also the case for single-factor potential risks. Specifically, the potential ecological risk indices of Cd and Hg vary greatly, ranging from no ecological risk to extremely high ecological risk, with average values of 33.45 and 65.90, respectively. The Er values of the other six HMs (Cr, Ni, Cu, Zn, Pb, As) are relatively low, with average values of 1.31, 3.45, 2.52, 1.05, 4.09, and 3.20, respectively, indicating low ecological risks. As can be seen from Table 1, Hg and Cd account for very little content in the sediments. However, due to their high toxicity, these two HMs occupy a much larger proportion in *RI* than other HMs and are the main potential pollution risk factors. Cd pollution is the most serious and brings extremely high ecological risks at DN01 and DN10, strong ecological risks at DN23 and DN25, and medium ecological risks at DN02, DN03, DN05, and DN15. The ecological risk of Hg at DN01 is as high as 735.94, and it is also extremely high at DN03, DN05, and DN23. Overall, the sampling points DN01, DN10 and DN23 are characterized by extremely high potential ecological risks, DN02, DN03 and DN05 carry strong ecological risks, DN17 and DN25 have medium ecological risks, and other sampling points have low risk levels. In Fig 5b, the distribution of toxicity risk coefficient *TRI* is slightly different. The overall assessment is that DN01, DN02, DN05, and DN23 have low toxicity, and other sampling points have no toxicity. Both risk indices show high risks at the tributary sampling points (DN01, DN02), tributary intersections (DN05), and river islands (DN23).

**Fig 5.** ***RI* values of heavy metal elements (a) and *TRI* distribution (b)**

In order to evaluate the health risks caused by heavy metal exposure, the hazard quotient (*HQ_ing_* and *HQ_dermal_*) and hazard index (*HI_ing_* and *HI_dermal_*) of individual heavy metal elements in the sediments of the Yihe River were calculated. This health assessment considers the health effects (Cr, Cd, As, Cu, Hg, Ni, Zn and Pb) and carcinogenic risks (As, Cr, Pb, Cd) of heavy metals. The calculated results are shown in Table 4. The results show that in all observation stations, the *HQ* values of all heavy metals in the two exposure pathways (direct ingestion and dermal contact) of sediments for adults and children were lower than 1. And the *HI* values for both children and adults were lower than the safety value of 1, indicating that the heavy metals in the study area have no non-carcinogenic risk to humans.

After calculating the exposure dose of heavy metals, the potential carcinogenic risk of exposure to Cr, Cd, Pb and As in sediments was calculated using the carcinogenic slope factor (Table 4). The average *CR_ing_* (ingestion carcinogenic risk) of sediments exposed to children through ingestion is Cr (2.35×10^-4^), Cd (9.88×10^-5^), Pb (2.26×10^-6^), and As (4.33×10^-5^), indicating that Cr has a high carcinogenic risk (*CR* > 10^-4^), and Cd, Pb, and As have acceptable carcinogenic risks (between 10^-6^ and 10^-4^). The potential carcinogenic risk *CR_dermal_* caused by dermal contact in children is less than 10^-6^, indicating that the carcinogenic risk to children through dermal contact with heavy metals in sediments is negligible. For children, the total carcinogenic risks are Cr (2.35×10^-4^), Cd (9.89×10^-6^), Pb (2.26×10^-6^), and As (4.33×10^-5^), among which the *CR* of Cr is higher than the threshold (1×10^-4^), indicating that Cr in sediment samples is an unacceptable carcinogenic risk for children. The carcinogenic risks *CR_ing_* and *CR_dermal_* caused by ingestion and dermal contact in adults are both within the acceptable range, and the total carcinogenic risk is 4.32×10^-5^, which is an acceptable carcinogenic risk.

**Table 4. Carcinogenic and non-carcinogenic risks of heavy metals in sediments of Yihe River**

|  |  | Metals | Cr | Cd | Pb | As | Ni | Cu | Zn | Hg |
| --- | --- | --- | --- | --- | --- | --- | --- | --- | --- | --- |
|  | Child |  |  |  |  |  |  |  |  |  |
| Non-cancer | *HQ_ing_* | min | 3.26E-02 | 3.21E-04 | 1.39E-01 | 3.43E-02 | 2.88E-03 | 7.32E-04 | 5.25E-04 | 2.14E-04 |
|  |  | max | 1.92E-01 | 7.82E-03 | 4.14E-01 | 3.38E-01 | 2.57E-02 | 1.20E-02 | 7.39E-03 | 1.88E-02 |
|  |  | mean | 9.39E-02 | 1.57E-03 | 1.90E-01 | 9.61E-02 | 1.04E-02 | 3.16E-03 | 2.50E-03 | 1.69E-03 |
|  | *HQ_dermal_* | min | 5.25E-05 | 5.14E-07 | 2.22E-04 | 5.48E-05 | 4.62E-06 | 1.17E-06 | 8.40E-07 | 3.43E-07 |
|  |  | max | 3.07E-04 | 1.25E-05 | 6.62E-04 | 5.41E-04 | 4.11E-05 | 1.92E-05 | 1.18E-05 | 3.01E-05 |
|  |  | mean | 1.50E-04 | 2.51E-06 | 3.04E-04 | 1.54E-04 | 1.66E-05 | 5.06E-06 | 4.00E-06 | 2.70E-06 |
|  | *HI_ing_* |  | 3.99E-01 | | | | | | | |
|  | *HI_dermal_* |  | 6.39E-04 | | | | | | | |
|  | *HI* |  | 4.00E-01 | | | | | | | |
| Cancer | *CR_ing_* | min | 8.20E-05 | 2.02E-06 | 1.65E-06 | 1.54E-05 | - | - | - | - |
|  |  | max | 4.79E-04 | 4.93E-05 | 4.92E-06 | 1.52E-04 | - | - | - | - |
|  |  | mean | 2.35E-04 | 9.88E-06 | 2.26E-06 | 4.32E-05 | - | - | - | - |
|  | *CR_dermal_* | min | 1.31E-07 | 3.24E-09 | 2.64E-09 | 2.47E-08 | - | - | - | - |
|  |  | max | 7.67E-07 | 7.88E-08 | 7.87E-09 | 2.43E-07 | - | - | - | - |
|  |  | mean | 3.76E-07 | 1.58E-08 | 3.62E-09 | 6.92E-08 | - | - | - | - |
|  | *CR* |  | 2.35E-04 | 9.89E-06 | 2.26E-06 | 4.33E-05 | - | - | - | - |
|  | *TCR* |  | 2.91E-04 | | | | | | | |
|  | ***Adults*** |  |  |  |  |  |  |  |  |  |
| Non-cancer | *HQ_ing_* | min | 3.52E-03 | 3.09E-04 | 7.84E-05 | 5.63E-05 | 3.44E-05 | 1.49E-02 | 3.67E-03 | 2.30E-05 |
|  |  | max | 2.05E-02 | 2.75E-03 | 1.28E-03 | 7.91E-04 | 8.38E-04 | 4.43E-02 | 3.62E-02 | 2.02E-03 |
|  |  | mean | 1.01E-02 | 1.11E-03 | 3.39E-04 | 2.68E-04 | 1.68E-04 | 2.03E-02 | 1.03E-02 | 1.81E-04 |
|  | *HQ_dermal_* | min | 1.40E-03 | 1.23E-04 | 3.13E-05 | 2.25E-05 | 1.37E-05 | 5.93E-03 | 1.46E-03 | 9.17E-06 |
|  |  | max | 8.20E-03 | 1.10E-03 | 5.12E-04 | 3.16E-04 | 3.34E-04 | 1.77E-02 | 1.44E-02 | 8.04E-04 |
|  |  | mean | 4.01E-03 | 4.43E-04 | 1.35E-04 | 1.07E-04 | 6.70E-05 | 8.12E-03 | 4.11E-03 | 7.20E-05 |
|  | *HI_ing_* |  | 4.28E-02 | | | | | | | |
|  | *HI_dermal_* |  | 1.71E-02 | | | | | | | |
|  | *HI* |  | 5.98E-02 | | | | | | | |
| Cancer | *CR_ing_* | min | 8.79E-06 | 2.17E-07 | 1.77E-07 | 1.65E-06 | - | - | - | - |
|  |  | max | 5.13E-05 | 5.28E-06 | 5.27E-07 | 1.63E-05 | - | - | - | - |
|  |  | mean | 2.52E-05 | 1.06E-06 | 2.42E-07 | 4.63E-06 | - | - | - | - |
|  | *CR_dermal_* | min | 3.51E-06 | 8.64E-08 | 7.05E-08 | 6.59E-07 | - | - | - | - |
|  |  | max | 2.05E-05 | 2.11E-06 | 2.10E-07 | 6.50E-06 | - | - | - | - |
|  |  | mean | 1.00E-05 | 4.22E-07 | 9.66E-08 | 1.85E-06 | - | - | - | - |
|  | *CR* |  | 3.52E-05 | 1.48E-05 | 3.39E-07 | 6.48E-06 | - | - | - | - |
|  | *TCR* |  | 4.32E-05 | | | | | | | |

# Discussion

The coefficient of variation (CV) is the ratio of standard deviation to the mean, which can eliminate the influence of unit difference and mean difference [6]. It can be used to analyze the impact of anthropogenic activities on environmental dynamics [8]. According to the CV, the level of variation can be divided into low variation (CV ≤ 0.20), medium variation (0.20 < CV ≤ 0.50), high variation (0.50 < CV ≤ 1.00), and extremely high variation (CV ≥ 1.00) [37]. Based on our calculations, the CVs of Ni, Cu, Zn, Cd, As, and Hg in the study area are all high and extremely high, and the CVs of Cr and Pb are less than 0.5, which are medium variations, indicating that the content of HMs in the sediments of the Yihe River is greatly affected by anthropogenic activities.

The global Moran's index is used to describe the overall spatial autocorrelation of heavy metal distribution in the entire study area. Its value range is -1 to 1, and global Moran's I > 0 indicates an clustering trend, and global Moran's I < 0 indicates a dispersed direction [38] .The spatial distribution characteristics of *RI* and *TRI* in the study area were evaluated using the global Moran's I, and the results are displayed in Fig 6. The results show that the values of global Moran's I are 0.106 and 0.332, respectively. The values are > 0 (P < 0.05), and the spatial distribution shows a clustering trend, indicating that *RI* and *TRI* may be affected by external factors. And high-high cluster in tributaries indicates that the heavy metal pollution level is higher there.

**Fig 6. Spatial autocorrelation results of *RI* and *TRI* in the study area.**

On a spatial scale, the distribution of HMs in Yihe River sediments varies greatly (Fig 2). HMs are concentrated in tributaries and near the central island of the Yihe River, indicating that human activities in this area strongly impact heavy metal accumulation. This is consistent with the *CF*, *PLI*, *I_geo_*, *TR*, and *TRI* evaluation results. The concentrations of Pb, As, Ni, Zn, and Cu are almost the highest in the Suhe River and Benghe River tributaries, which may be related to the geographical location: there are frequent human activities along these tributaries. The Suhe River and Benghe River are close to residential and transportation areas and are seriously affected by man-made impacts. For example, pollutants discharged from the industrial park in the western part of the study area enter the Suhe River and Benghe River, which is also the reason for the high concentration in the tributaries. The concentration of HMs at DN05 remains at a high level, probably because this point is located at the intersection of three tributaries and is greatly affected by tributary pollution. It is worth noting that at sampling point DN23, the concentration of HMs is significantly higher than at adjacent sampling points upstream and downstream. DN23 is located downstream of Hexin Island (Yihe North Island), where the water flow is slow and HMs are easy to accumulate, and this point may be affected by the Xiaobudong Rubber Dam. Rubber dams can reduce the water flow rate and thus have a blocking effect; the reduced flow rate accelerates the deposition of suspended matter in the water. This point is closest to the rubber dam upstream, and the sedimentation effect may be more obvious. In terms of time, compared with the data measured in 2009 (Table 5) [18], the Hg and Cd contents of the Suhe River increased, which may be related to the development and utilization of the North and South Streets of the Suhe River. In comparison, the contents of As, Cu, Cr, Pb, and Cd in the Liuqinghe River decreased significantly. The local government implemented the treatment of the Liuqinghe River in 2017, which may be the reason for this decrease. Meanwhile, the contents of As, Cu, Cr, Pb, Hg, and Cd in the main stream of the Yihe River also decreased significantly. Normally, the heavy metal content in sediments is hundreds of times or even more than that in water bodies. Compared with the heavy metal content in the water bodies of the Yihe River in historical studies [39,40], the heavy metal content in the sediments of the Yihe River is several to 100 times that of the heavy metal content in historical water bodies, indicating that the heavy metal content in the Yihe River has decreased since 2013. The decrease is closely related to the local government's management and protection of the Yihe River.

**Table 5. Comparison of heavy metal concentrations in historical sediments of the main and tributary rivers of the Yihe River (mg/kg)**

| Years | River | As | Cd | Cr | Cu | Hg | Pb |
| --- | --- | --- | --- | --- | --- | --- | --- |
| 2009 | Benghe River | 5.21 | 0.25 | 74.23 | 34.68 | 0.07 | 37.12 |
|  | Suhe River | 7.09 | 0.23 | 70.35 | 32.15 | 0.23 | 36.68 |
|  | Liuqinghe River | 5.97 | 1.56 | 84.64 | 50.63 | 0.06 | 59.28 |
|  | Main stream of Yihe River | 7.09 | 0.27 | 67.88 | 46.29 | 0.17 | 40.78 |
| 2023 | Benghe River | 5.73 | 0.18 | 69.70 | 29.34 | 0.05 | 28.13 |
|  | Suhe River | 7.39 | 0.45 | 67.63 | 37.50 | 0.44 | 45.28 |
|  | Liuqinghe River | 2.35 | 0.14 | 33.03 | 14.97 | 0.07 | 24.25 |
|  | Main stream of Yihe River | 1.00 | 0.03 | 31.63 | 3.17 | 0.01 | 17.64 |

Different age groups have different activity patterns and thus different exposure parameters. Children spend significantly more time outdoors than adults and may be more exposed to pollution sources (higher exposure time and frequency). Therefore, children should have a higher exposure risk. In this study, Cr in sediment samples posed an unacceptable carcinogenic risk to children, with the main route of exposure being ingestion. For adults, heavy metals pose no significant carcinogenic and non-carcinogenic risks. This may be related to children's living habits and their body's ability to excrete heavy metals.

We compared the heavy metal content found in Yihe River sediments with rivers in other regions analyzed in the literature (Table 6). Compared with the Yishui County area in the upper reaches of the Yihe River, the heavy metal content in Linyi City is significantly lower, which is related to the developed industry and agriculture in Yishui County in the upper reaches of the Yihe River. Statistics show that the comprehensive energy consumption of industries above designated size in Yishui County is 1.6 times that of urban areas, and the use of agricultural fertilizers is 1.25 times that of urban areas, resulting in a higher heavy metal input to the Yishui County area. Comparing the Yihe River with other rivers in Shandong Province, the heavy metal content in the Xiaoqinghe River is higher. This may be closely related to the concentration of industries in the Xiaoqinghe River Basin. Historically, petrochemical, steel, chemical and other enterprises have long been present in the Xiaoqinghe River Basin, and HMs were imported from industrial sources. These were a major source of HMs in the Xiaoqinghe River [41]. The heavy metal contents in the Yihe River and the Daguhe River are similar, which may be related to the fact that the Yihe River and the Daguhe River may be affected by the same type and degree of agriculture and industries. The Yihe River is a typical river in northern China, and a comparative analysis was conducted between it and the rivers in southern China. The Yihe River has higher heavy metal content than the Xiangxi River in Hubei Province, which may be related to different climatic conditions. The Yihe River has a temperate monsoon climate, with an average annual rainfall of 800~848 mm, while Xiangxi River has a subtropical monsoon climate, with an average annual rainfall of 916~1080 mm [42]. The differences in rainfall may have an impact on heavy metal content in sediments. When precipitation increases, on the one hand, it will dilute the concentration of HMs; on the other hand, the hydrodynamic conditions in the river will change, and the HMs in the sediment will re-enter the water body, reducing the content of HMs in the sediment. The Zn and Cu concentrations in Qinjiang River, Guangxi Province are significantly higher than those in Yihe River, and the contents of other HMs are similar to those in the Yihe River. This may be related to the mining of local zinc ore and manganese ore in Guangxi Province.

**Table 6.** **Comparison of average heavy metal concentrations in Yihe River sediments analyzed in this study and the relevant literature (mg/kg)**

| River | Province | Heavy metal concentration in sediment (mg/kg) | | | | | | | | References |
| --- | --- | --- | --- | --- | --- | --- | --- | --- | --- | --- |
|  |  | Cr | Ni | Cu | Zn | Cd | Pb | As | Hg |  |
| Yihe River | Shandong Province | 36.72 | 16.22 | 9.89 | 58.73 | 0.12 | 20.79 | 2.26 | 0.04 | This study |
| Yihe River Upstream Yishui County | Shandong Province | 131.79 | 42.29 | 24.29 | 165.59 | 0.27 | 21.18 | 2.13 | 0.66 | [43] |
| Daguhe River | Shandong Province | 56.81 | - | 18.42 | 69.01 | 0.083 | 13.51 | 9.84 | - | [44] |
| Xiaoqinghe River | Shandong Province | 171.61 | 31.99 | 62.77 | 314.25 | 0.62 | 49.06 | 11.10 | - | [41] |
| Xiangxi River | Hubei Province | - | 6.2 | 3.21 | 2.4 | 0.17 | 10.59 | 0.65 | 0.05 | [45] |
| Qinjiang River | Guangxi Province | 29.78 | 17.82 | 65.45 | 112.49 | 0.02 | 22.99 | - | - | [46] |

Pearson correlation analysis is widely used to determine the possible sources and relationships of HMs in the environment [47]. The correlation between HMs may reflect the sources and migration pathways of heavy metal elements [48, 49]. Table 7 presents the calculated sediment correlation analysis matrix. The data reveal that, except for Cr~Cd that has no significant correlation, other HMs have a significant positive correlation. Among them, there is a strong correlation between Ni, Cu, Zn, Cr, and As, and their spatial distributions are similar (Fig 2), indicating that they may come from the same source. There is no significant correlation between Ni and pH, and the remaining seven HMs have a significant negative correlation with pH, indicating that increasing pH may reduce the content of HMs in sediments. Sediment organic matter (SOM) can affect the ecotoxicity, environmental changes and geochemical status of trace metals in sediments [50]. The organic matter in the Yihe River sediments has a significant positive correlation with eight HMs and a high correlation with HMs except Cd. Among them, the correlation with Cu (0.933) and As (0.935) is the highest, indicating that organic matter in the Yihe River sediments has a significant positive correlation with HMs. The content or organic matter is closely related to HM concentration, thus they may have the same source or migration pathway.

**Table 7.** **Pearson correlation analysis matrix of HMs in Yihe River sediments**

|  | Cr | Ni | Cu | Zn | Cd | Pb | As | Hg | pH | SOM |
| --- | --- | --- | --- | --- | --- | --- | --- | --- | --- | --- |
| Cr | 1 |  |  |  |  |  |  |  |  |  |
| Ni | 0.833 ^**^ | 1 |  |  |  |  |  |  |  |  |
| Cu | 0.872 ^**^ | 0.923 ^**^ | 1 |  |  |  |  |  |  |  |
| Zn | 0.754 ^**^ | 0.883 ^**^ | 0.924 ^**^ | 1 |  |  |  |  |  |  |
| Cd | 0.376 | 0.442^*^ | 0.581^**^ | 0.715^**^ | 1 |  |  |  |  |  |
| Pb | 0.700^**^ | 0.765^**^ | 0.899^**^ | 0.870^**^ | 0.796^**^ | 1 |  |  |  |  |
| As | 0.884^**^ | 0.890^**^ | 0.967^**^ | 0.852^**^ | 0.461^*^ | 0.815^**^ | 1 |  |  |  |
| Hg | 0.585^**^ | 0.574 ^**^ | 0.740 ^**^ | 0.616 ^**^ | 0.552 ^**^ | 0.866 ^**^ | 0.680 ^**^ | 1 |  |  |
| pH | -0.488 ^*^ | -0.374 | -0.609 ^**^ | -0.527 ^**^ | -0.667 ^**^ | -0.732 ^**^ | -0.554 ^**^ | -0.756 ^**^ | 1 |  |
| SOM | 0.827 ^**^ | 0.833 ^**^ | 0.933 ^**^ | 0.783 ^**^ | 0.449 ^*^ | 0.836 ^**^ | 0.935 ^**^ | 0.753 ^**^ | -0.611 ^**^ | 1 |

Notes:

*Correlation is significant at the 0.05 level (two-tailed).

**Correlation is significant at the 0.01 level (two-tailed).

The HM loads measured in the sediment samples of the Yihe River were classified using the Varimax rotation method. PCA identified two principal components (PCs), which explained 89.6% of the total variance, indicating that there are different controlling factors or sources of HMs in the Yihe River sediments. The eigenvalues, explained variance, percentage of variance extracted by different components, and factor loadings of different variables are shown in Table 8. PC1 accounts for 54.22% of the total variance, with high loadings for Cr (0.913), Ni (0.901), Cu (0.864), and As (0.906), and medium loadings for Zn (0.73), which is consistent with the high correlation between these metals discussed above. Previous studies have suggested that Cr and Ni are elements of natural origin, mainly affected by natural factors such as the soil parent materials [17, 51]. As is an important component in insecticides, herbicides, fungicides, desiccants, defoliants, and animal feed additives, and Cu may originate from the excessive application of pesticides, herbicides or fertilizers in farmland [45, 52, 53]. The Yihe River Basin is an important area of vegetable production and fruit and flower source for Linyi City. Agricultural activities are frequent along the upper reaches of the Yihe River, and there are dense parks and squares along the river and its tributaries. A large amount of pesticides and herbicides used in agricultural activities are not completely absorbed; the HMs contained therein flow into the Yihe River and its tributaries with surface runoff and are deposited in sediments through physical adsorption or chemical precipitation. Therefore, PC1 is a mixed source of agricultural activities and natural sources.

PC2 explains 35.56% of the total variance, of which Cd (0.936) reaches high load, Pb (0.794) and Hg (0.682) are in medium loads. PC2 can be represented as a mixed source of traffic and industrial pollution. Studies have shown that environmental Pb is closely related to the combustion of coal, oil and other fossil fuels, and is generated by paint, shipping and industrial wastewater discharge [6, 54-56]. According to the Linyi Statistical Yearbook 2023, the number of civil motor vehicles in Linyi City reached 3,527,516 in 2022, and the traffic along the Yihe River was well developed, with a multitude of cross-river roads and riverside roads. Gasoline residues and automobile exhaust were a major source of Pb in the study area. Hg mainly comes from the combustion of fossil fuels such as coal. In many industrial production processes, Hg is widely used as additives and catalysts [57]. Cd mainly originates from metal smelting and electroplating. In 2022, the total industrial output value of Linyi City accounted for 33.1% of the total GDP, and a total of 20,402,545 tons of standard coal were consumed. Among them, the standard coal consumed by the metal smelting and rolling processing industry and the metal preparation industry accounted for about 40.15% of the total consumption, which may have been the source of Hg and Cd in sediments.

**Table 8.** **Characteristic values and cumulative contribution rates of heavy metals in sediments**

| Total ingredients explained | | | | | | | | | | | |
| --- | --- | --- | --- | --- | --- | --- | --- | --- | --- | --- | --- |
| Element | Initial eigenvalue |  |  | Extract the sum of squared loads |  |  | Rotating load sum of squares |  |  | Rotated component matrix | |
|  | Total | Contribution rate | Grand total% | total | Contribution rate | Grand total% | Total | Contribution rate | Grand total% | PC1 | PC2 |
| Cr | 6.271 | 78.388 | 78.388 | 6.271 | 78.388 | 78.388 | 4.338 | 54.224 | 54.224 | 0.913 | 0.215 |
| Me | 0.896 | 11.196 | 89.584 | 0.896 | 11.196 | 89.584 | 2.829 | 35.36 | 89.584 | 0.901 | 0.307 |
| Cu | 0.481 | 6.013 | 95.598 |  |  |  |  |  |  | 0.864 | 0.489 |
| Zn | 0.181 | 2.268 | 97.865 |  |  |  |  |  |  | 0.73 | 0.593 |
| Cd | 0.09 | 1.12 | 98.985 |  |  |  |  |  |  | 0.15 | 0.936 |
| Pb | 0.049 | 0.617 | 99.602 |  |  |  |  |  |  | 0.587 | 0.794 |
| As | 0.023 | 0.282 | 99.884 |  |  |  |  |  |  | 0.906 | 0.354 |
| Hg | 0.009 | 0.116 | 100 |  |  |  |  |  |  | 0.475 | 0.682 |

The APCS-MLR method can overcome the weakness of PCA being insufficient to provide quantitative information based on the contribution of each source type. Based on the PCA results, the APCS-MLR receptor model was used to obtain the contribution of HMs from various sources in the sediments of the Yihe River, and the results are shown in Table 9. The contribution of PC1 to different HMs ranges from 23.49% (Cd) to 88.99% (Cr), and was 84.80% (Ni), 82.92% (As), 77.03% (Cu), and 70.07% (Zn), respectively. PC2 accounts for 76.51% of Cd, 41.56% of Pb, and 43.09% of Hg. 67.83% of HMs in sediments came from mixed sources of agricultural activities and natural sources, and the average contribution percentage of traffic pollution and industrial sources was 32.17%. According to the multiple regression results, the goodness of fit R^2^ is between 0.663 and 0.984, and the measured value to predicted value (M/P) of all elements are equal to 1, indicating that the constructed APCS-MLR receptor model is suitable for estimating the source distribution of HMs in sediment samples from different land use areas in the Yihe River, and the evaluation results are relatively reliable.

**Table 9.** **APCS-MLR calculation of heavy metal source contribution rate (%) and predicted ratio (M/P)**

|  | PC1 | PC2 | M/P | R^2^ |
| --- | --- | --- | --- | --- |
| Cr | 88.99 | 11.01 | 1.0 | 0.869 |
| Ni | 84.80 | 15.20 | 1.0 | 0.897 |
| Cu | 77.03 | 22.97 | 1.0 | 0.984 |
| Zn | 70.07 | 29.93 | 1.0 | 0.884 |
| cd | 23.49 | 76.51 | 1.0 | 0.890 |
| Pb | 58.44 | 41.56 | 1.0 | 0.972 |
| As | 82.92 | 17.08 | 1.0 | 0.941 |
| Hg | 56.91 | 43.09 | 1.0 | 0.663 |

In response to the problem of heavy metal pollution in the sediments of the Yihe River, it is crucial to implement effective management and control measures based on actual conditions. We proposed some ideas for the prevention and control of heavy metals in Yihe River sediments. (1) Strengthen the supervision and performance evaluation of the mechanism for eliminating obsolete production capacity, and regularly disclose and update the negative list of enterprises that need to be withdrawn within a time limit. For emerging industries involving heavy metal emissions, implement the strategy of "equal replacement" or "reduction replacement" to control the total amount of emissions [58]. (2) Strengthen the supervision of key polluting industries, especially the environmental governance of high-pollution industries such as plastic processing and electroplating, to ensure that pollution is effectively controlled. In addition, actively promote the circular economy and clean production model to promote efficient resource utilization and environmental protection. (3) In order to reduce the emission of heavy metals in motor vehicle exhaust, it is necessary to formulate and implement strict emission management policies. Vehicles that do not meet emission standards should be gradually restricted and eliminated. Vehicle owners should be encouraged to install exhaust treatment devices to improve emission purification efficiency; and policy incentives such as car purchase subsidies should be used to promote the popularization of electric vehicles; and citizens should be encouraged to take public transportation to effectively reduce the content of heavy metals in atmospheric deposition sources. (4) The application of chemical fertilizers should adhere to scientific and rational principles, aiming to enhance their efficiency in nutrient delivery. It is imperative to boost the utilization ratio of these fertilizers and rigorously ban the usage of any fertilizer that contains excessively high levels of heavy metals. Reuse crop straw, livestock and poultry manure and other agricultural waste into organic fertilizer or biomass energy, reducing the use of chemical fertilizers and heavy metal pollution. The above measures can effectively solve the problem of heavy metal pollution in the sediments of the Yihe River.

# Conclusion

The average concentrations of eight HMs in the sediments of the Linyi urban section of the Yihe River were Cr (36.72 mg/kg^−1^), Ni (16.22 mg/kg^−1^), Cu (9.89 mg/kg^−1^), and Zn (58.73 mg/kg^−1^), Cd (0.12 mg/kg^−1^), Pb (20.79 mg/kg^−1^) , As (2.26 mg/kg^−1^) and Hg (0.04 mg/kg^−1^).

The spatial distribution of HMs mainly shows that the tributaries Suhe River (DN01), Benghe River (DN02), and Liuqinghe River (DN03) are seriously polluted. HMs also exhibit higher concentrations in the sediments at the intersection of the three tributaries and the North Island of Yihe River.

HMs in the sediments of the tributaries of Suhe River, Benghe River, Liuqinghe River and the North Island of Yihe River have low toxicity risk and strong ecological risk. HMs are in a pollution-free state in most areas of the main stream of Yihe River, with low ecological risk and no toxicological risk. Hg and Cd are the main contributors to the ecological risks.

HMs have no non-carcinogenic risk to humans. Cr is the main carcinogenic factor and has an unacceptable carcinogenic risk to children. As and Cd have an acceptable carcinogenic risk to both adults and children.

HMs in the sediments of the Linyi urban section of the Yihe River mainly come from two aspects: Cr, Ni, Cu, Zn, and As mainly originate from natural sources and agricultural activities, with a contribution rate of 67.83%; Cd, Pb and Hg are mainly produced by transportation and industry, with an average proportion of 32.17%. These research results provide an important reference for pollution control and planning prevention in the Yihe River Basin.

# Supporting information

S1 Table. Exposure factors for human health risk assessment.

S2 Table. The distribution of specific heavy metals.

S3 Table. *PLI* values calculated at each sampling point.

S4 Table. *I_geo_* values calculated at each sampling point.

S5 Table. *RI* values calculated at each sampling point.

S6 Table. *TRI* values calculated at each sampling point.

# Acknowledgments

The authors sincerely thank all the staff for their diligent assistance in the sampling process. Their unwavering support throughout the sampling phase have been crucial for the quality and integrity of our research. And we would like to express our sincere appreciation to The Seventh Geological Bridge of Shandong Provincial Bureau of Geology and Mineral for providing us with excellent experimental conditions. Their facilities and resources have been invaluable in conducting our analyses and obtaining reliable results.

# References

1. Dai L, Wang L, Li L, Liang T, Zhang Y, Ma C, et al. Multivariate geostatistical analysis and source identification of heavy metals in the sediment of Poyang Lake in China. Sci Total Environ. 2018;621:1433-44. doi: 10.1016/j.scitotenv.2017.10.085. PMID: [29056381](https://pubmed.ncbi.nlm.nih.gov/29056381/).
2. Haddout S, Priya KL, Casila JCC, Samad B. Assessment of heavy metal contamination in the surficial sediments from the Sebou River Estuary, Morocco. Environmental Forensics. 2021;23(1-2):23-31. doi: 10.1080/15275922.2021.1887970.
3. Islam MS, Hossain MB, Matin A, Islam Sarker MS. Assessment of heavy metal pollution, distribution and source apportionment in the sediment from Feni River estuary, Bangladesh. Chemosphere. 2018;202:25-32. doi: 10.1016/j.chemosphere.2018.03.077. PMID: [29554504](https://pubmed.ncbi.nlm.nih.gov/29554504/).
4. Fadlillah LN, Utami S, Rachmawati AA, Jayanto GD, Widyastuti M. Ecological risk and source identifications of heavy metals contamination in the water and surface sediments from anthropogenic impacts of urban river, Indonesia. Heliyon. 2023;9(4):e15485. doi: 10.1016/j.heliyon.2023.e15485. PMID: [37151694](https://pubmed.ncbi.nlm.nih.gov/37151694/).
5. Proshad R, Kormoker T, Abdullah Al M, Islam MS, Khadka S, Idris AM. Receptor model-based source apportionment and ecological risk of metals in sediments of an urban river in Bangladesh. J Hazard Mater. 2022;423(Pt A):127030. doi: 10.1016/j.jhazmat.2021.127030. PMID: [34482078](https://pubmed.ncbi.nlm.nih.gov/34482078/).
6. Wang J, Wu J, Zhan S, Jin M, Zhou J, Shen B, et al. Spatial enrichment assessment, source identification and health risks of potentially toxic elements in surface sediments, Central Asian countries. Journal of Soils and Sediments. 2021;21(12):3906-16. doi: 10.1007/s11368-021-03061-3.
7. Algul F, Beyhan M. Concentrations and sources of heavy metals in shallow sediments in Lake Bafa, Turkey. Sci Rep. 2020;10(1):11782. doi: 10.1038/s41598-020-68833-2. PMID: [32678245](https://pubmed.ncbi.nlm.nih.gov/32678245/).
8. Liu P, Wu Q, Hu W, Tian K, Huang B, Zhao Y. Comparison of heavy metals in riverine and estuarine sediments in the lower Yangtze River: Distribution, sources, and ecological risks. Environmental Technology & Innovation. 2023;30. doi: 10.1016/j.eti.2023.103076.
9. Shen F, Mao L, Sun R, Du J, Tan Z, Ding M. Contamination Evaluation and Source Identification of Heavy Metals in the Sediments from the Lishui River Watershed, Southern China. Int J Environ Res Public Health. 2019;16(3). doi: 10.3390/ijerph16030336. PMID: [30691076](https://pubmed.ncbi.nlm.nih.gov/30691076/).
10. Li H-S, Gu Y-G, Liang R-Z, Wang Y-S, Jordan RW, Wang L-G, et al. Heavy metals in riverine/estuarine sediments from an aquaculture wetland in metropolitan areas, China: Characterization, bioavailability and probabilistic ecological risk. Environmental Pollution. 2023;324:121370.
11. Xie H, Yang X, Xu J, Zhong D. Heavy metals pollution and potential ecological health risk assessment in the Yangtze River reaches. Journal of Environmental Chemical Engineering. 2023;11(2). doi: 10.1016/j.jece.2023.109489.
12. Abebe Y, Alamirew T, Whitehead P, Charles K, Alemayehu E. Spatio-temporal variability and potential health risks assessment of heavy metals in the surface water of Awash basin, Ethiopia. Heliyon. 2023;9(5):e15832. doi: 10.1016/j.heliyon.2023.e15832. PMID: [37215855](https://pubmed.ncbi.nlm.nih.gov/37215855/).
13. Radhakrishnan N, Taneja S, Ambastha S, Pipil H, Haritash AK. Heavy metal profile, mobility, and source characterization in size-fractionated bed-sediments of River Ganga, India. Mar Pollut Bull. 2023;188:114650. doi: 10.1016/j.marpolbul.2023.114650. PMID: [36758312](https://pubmed.ncbi.nlm.nih.gov/36758312/).
14. Dhiman A, Ramanthan AL, Macklin M, Yadav S, Kushwaha S, Mudbhatkal A, et al. Heavy metal distribution in various environmental matrices and their risk assessment in Ganga River Basin, India. Human and Ecological Risk Assessment: An International Journal. 2022;29(2):621-50. doi: 10.1080/10807039.2022.2159785.
15. Ezewudo BI, Mgbenka BO, Islam MS, Proshad R, Odo GE. Appraisal of metal contamination in sediments of lower reaches of Niger River, Nigeria, using contamination indices and sediment quality guidelines. International Journal of Environmental Analytical Chemistry. 2021;103(11):2616-35. doi: 10.1080/03067319.2021.1895140.
16. Ustaoğlu F. Ecotoxicological risk assessment and source identification of heavy metals in the surface sediments of Çömlekci stream, Giresun, Turkey. Environmental Forensics. 2020;22(1-2):130-42. doi: 10.1080/15275922.2020.1806148.
17. Chen Y, Guo Y, Lv Z. Pollution characteristics and potential ecological risk of heavy metals in the surface sediments from the Linyi section of Yi River. Journal of Capital Normal University(Natural Science Edition). 2021;42(02):66-72. doi: 10.19789/j.1004-9398.2021.02.010.(in Chinese)
18. Hu Y, Zhao H, Xu Q, Feng X, Ru S. Distribution and ecological risk assessment of heavy metals in the surface sediment of Xiaobudong Rubber Dam in the Yihe River. Reserach of Environmental Sciences. 2012;25(3):304-10.(in Chinese)
19. Zhuo L, Du F, Yin Y, Cui Z. Distribution and ecological risk assessment for heavy metals in superficial sediment of Yihe River. Journal of Zaozhuang University. 2016;33(2):82-6.(in Chinese)
20. Li B, Sun C, Zhang Z, Liu Y. Effects of Xiaobudong Rubber Dam on the distribution of sediment heavy metals in Yihe river at Linyi City, Shandong Province. Journal of Soil and Water Conservation. 2014;28(4):243-6.(in Chinese)
21. Li X, Shen H, Zhao Y, Cao W, Hu C, Sun C. Distribution and Potential Ecological Risk of Heavy Metals in Water, Sediments, and Aquatic Macrophytes: A Case Study of the Junction of Four Rivers in Linyi City, China. Int J Environ Res Public Health. 2019;16(16). doi: 10.3390/ijerph16162861. PMID: [31405094](https://pubmed.ncbi.nlm.nih.gov/31405094/).
22. Hakanson L. An ecological risk index for aquatic pollution control. A sedimentological approach. Water research. 1980;14(8):975-1001.
23. Harikrishnan N, Ravisankar R, Chandrasekaran A, Suresh Gandhi M, Kanagasabapathy KV, Prasad MVR, et al. Assessment of Heavy Metal Contamination in Marine Sediments of East Coast of Tamil Nadu Affected by Different Pollution Sources. Mar Pollut Bull. 2017;121(1-2):418-24. doi: 10.1016/j.marpolbul.2017.05.047. PMID: [28633945](https://pubmed.ncbi.nlm.nih.gov/28633945/).
24. Tomlinson D, Wilson J, Harris C, Jeffrey D. Problems in the assessment of heavy-metal levels in estuaries and the formation of a pollution index. Helgoländer meeresuntersuchungen. 1980;33:566-75.
25. Wang J, Song J, Xie R, Li Y, Xu M, Lu J, et al. Source identification of heavy metals in surface sediments from a river in Anhui, China. Environmental Forensics. 2020;21(2):167-75. doi: 10.1080/15275922.2020.1728439.
26. Müller G. Index of geoaccumulation in sediments of the Rhine River. 1969;108–118.
27. Turekian KK, Wedepohl KH. Distribution of the elements in some major units of the earth's crust. Geological society of America bulletin. 1961;72(2):175-92.
28. Ustaoğlu F, Islam MS. Potential toxic elements in sediment of some rivers at Giresun, Northeast Turkey: A preliminary assessment for ecotoxicological status and health risk. Ecological Indicators. 2020;113. doi: 10.1016/j.ecolind.2020.106237.
29. Islam MS, Shammi RS, Jannat R, Kabir MH, Islam MS. Spatial distribution and ecological risk of heavy metal in surface sediment of Old Brahmaputra River, Bangladesh. Chemistry and Ecology. 2022. doi: 10.21203/rs.3.rs-1812855/v1.
30. Samal P, Singarasubramanian SR, Manoj MC, Srivastava J, Dsouza N, Balakrishna K, et al. Heavy metal contamination assessment and its associated human health risk evaluation in the Mahanadi River sediments, India. International Journal of Environmental Science and Technology. 2022;20(10):10673-94. doi: 10.1007/s13762-022-04630-w.
31. Hossain MB, Semme SA, Ahmed ASS, Hossain MK, Porag GS, Parvin A, et al. Contamination levels and ecological risk of heavy metals in sediments from the tidal river Halda, Bangladesh. Arabian Journal of Geosciences. 2021;14(3). doi: 10.1007/s12517-021-06477-w.
32. Zhao X-M, Yao L-A, Ma Q-L, Zhou G-J, Wang L, Fang Q-L, et al. Distribution and ecological risk assessment of cadmium in water and sediment in Longjiang River, China: Implication on water quality management after pollution accident. Chemosphere. 2018;194:107-16.
33. Liu Y, Jiang R, Kuang W, Lin C, Sun X, Chen J, et al. Characterization, source identification, risk assessment of potentially toxic elements (PTEs) in the surface water and sediment of the Beibu Gulf, China. Mar Pollut Bull. 2023;191:114905. doi: 10.1016/j.marpolbul.2023.114905. PMID: [3706213](https://pubmed.ncbi.nlm.nih.gov/3706213/).
34. EPA U. Risk Assessment Guidance for Superfund: pt. A. Human health evaluation manual (vol. 1). Office of Emergency and Remedial Response, US Environmental Protection …; 1989.
35. Magni LF, Castro LN, Rendina AE. Evaluation of heavy metal contamination levels in river sediments and their risk to human health in urban areas: A case study in the Matanza-Riachuelo Basin, Argentina. Environ Res. 2021;197:110979. doi: 10.1016/j.envres.2021.110979. PMID: [33711323](https://pubmed.ncbi.nlm.nih.gov/33711323/).
36. Jolly YN, Rakib MRJ, Kumar R, Islam ARMT, Rabby A, Mamun KM, et al. Deciphering the source of heavy metals in industrially affected river sediment of Shitalakshya river, Bangladesh, and potential ecological and health implications. Journal of Hazardous Materials Advances. 2023;10. doi: 10.1016/j.hazadv.2023.100268.
37. Cai Y, Wei J, Zhang S, Wang X, Zhang Z, Gao J. Spatial Risk Assessment and Source Identification of Heavy Metals in Riverine Sediments of Lake Chaohu Basin, China. Ecosystem Health and Sustainability. 2023;9. doi: 10.34133/ehs.0040.
38. Zhang Y, Song B, Zhou Z. Pollution assessment and source apportionment of heavy metals in soil from lead – Zinc mining areas of south China. Journal of Environmental Chemical Engineering. 2023;11(2). doi: 10.1016/j.jece.2023.109320.
39. Huo X, Hu C, Li X. Investigation of heavy metal pollution in the urban section of Yihe River in Linyi. Journal of Green Science and Technology. 2017,(22):25-26.(in Chinese)
40. Zhao Z, Liu Y, Sun C, Li B. Distribution and Pollution Assessment of Heavy Metals in the Water of Yihe River in Shandong Province. Journal of Anhui Agricultural Sciences. 2014,(08):2429-2431.
41. Dai J, Zhao Y, Li X, Wang Z, Liu Y, Xu Y, et al. Pollution assessment and source analysis of heavy metals and nutrients in surface sediments of Jinan section of Xiaoqing River. Environmental Chemistry. 2021;40(6):1795-807.(in Chinese)
42. Liu H, Ren H, Zhang P, Lu X, Zhang X. Analysis of characteristics of variation and abrup change of precipitation in Xiangxi River watershed over the past 60 years. Research of Soil and Water Conversation. 2015;22(4):5.
43. Hu X, Li B, Yan Y, Wang Z, X S, editors. Distribution of heavy metals in surface sediments and their relevance to release risk before rubber dam for river of Yishui city section. Proceedings of the 2021 Science and Technology Annual Conference of the Chinese Society of Environmental Science - Environmental Engineering Technology Innovation and Application; 2021. Tianjin, China.(in Chinese)
44. Sun H, Hu H, Gao P, Chen G, Jiao J, Yang Z. Ecological risk assessment of heavy metals in the surface sediments in dagu river. Transactions of Oceanology and Limnology. 2020;05:95-104.(in Chinese)
45. Xiong B, Li R, Johnson D, Luo Y, Xi Y, Ren D, et al. Spatial distribution, risk assessment, and source identification of heavy metals in water from the Xiangxi River, Three Gorges Reservoir Region, China. Environmental Geochemistry and Health. 2020;43(2):915-30. doi: 10.1007/s10653-020-00614-2.(in Chinese)
46. Zhang S, Chen B, Du J, Wang T, Shi H, Wang F. Distribution, Assessment, and Source of Heavy Metals in Sediments of the Qinjiang River, China. Int J Environ Res Public Health. 2022;19(15). doi: 10.3390/ijerph19159140. PMID: [35897501](https://pubmed.ncbi.nlm.nih.gov/35897501/).
47. Xiao H, Shahab A, Xi B, Chang Q, You S, Li J, et al. Heavy metal pollution, ecological risk, spatial distribution, and source identification in sediments of the Lijiang River, China. Environ Pollut. 2021;269:116189. doi: 10.1016/j.envpol.2020.116189. PMID: [33288295](https://pubmed.ncbi.nlm.nih.gov/33288295/).
48. Ke X, Gui S, Huang H, Zhang H, Wang C, Guo W. Ecological risk assessment and source identification for heavy metals in surface sediment from the Liaohe River protected area, China. Chemosphere. 2017;175:473-81. doi: 10.1016/j.chemosphere.2017.02.029. PMID: [28242463](https://pubmed.ncbi.nlm.nih.gov/28242463/).
49. Proshad R, Kormoker T, Islam S. Distribution, source identification, ecological and health risks of heavy metals in surface sediments of the Rupsa River, Bangladesh. Toxin Reviews. 2019;40(1):77-101. doi: 10.1080/15569543.2018.1564143.
50. Armid A, Shinjo R, Zaeni A, Sani A, Ruslan R. The distribution of heavy metals including Pb, Cd and Cr in Kendari Bay surficial sediments. Mar Pollut Bull. 2014;84(1-2):373-8. doi: 10.1016/j.marpolbul.2014.05.021. PMID: [24880681](https://pubmed.ncbi.nlm.nih.gov/24880681/).
51. Liu X, Chen S, Yan X, Liang T, Yang X, El-Naggar A, et al. Evaluation of potential ecological risks in potential toxic elements contaminated agricultural soils: Correlations between soil contamination and polymetallic mining activity. J Environ Manage. 2021;300:113679. doi: 10.1016/j.jenvman.2021.113679. PMID: [34509816](https://pubmed.ncbi.nlm.nih.gov/34509816/).
52. Xie Y, Huo X, Hu C, Tao Y. Contamination, Ecological Risk and Source Apportionment of Heavy Metals in the Surface Sediments in the Hailar River, the Upper Source of the Erguna River between China and Russia. Sustainability. 2023;15(4). doi: 10.3390/su15043655.
53. Zhuang Q, Li G, Liu Z. Distribution, source and pollution level of heavy metals in river sediments from South China. Catena. 2018;170:386-96. doi: 10.1016/j.catena.2018.06.037.
54. Islam MS, Nakagawa K, Abdullah-Al-Mamun M, Siddique MAB, Berndtsson R. Toxicity and source identification of pollutants in an urban river in Bangladesh. Environmental Earth Sciences. 2023;82(6). doi: 10.1007/s12665-023-10812-7.
55. Li W, Qian H, Xu P, Zhang Q, Chen J, Hou K, et al. Distribution characteristics, source identification and risk assessment of heavy metals in surface sediments of the Yellow River, China. Catena. 2022;216. doi: 10.1016/j.catena.2022.106376.
56. Yavar Ashayeri N, Keshavarzi B. Geochemical characteristics, partitioning, quantitative source apportionment, and ecological and health risk of heavy metals in sediments and water: A case study in Shadegan Wetland, Iran. Mar Pollut Bull. 2019;149:110495. doi: 10.1016/j.marpolbul.2019.110495. PMID: [31421566](https://pubmed.ncbi.nlm.nih.gov/31421566/).
57. Li X, Bing J, Zhang J, Guo L, Deng Z, Wang D, et al. Ecological risk assessment and sources identification of heavy metals in surface sediments of a river-reservoir system. Sci Total Environ. 2022;842:156683. doi: 10.1016/j.scitotenv.2022.156683. PMID: [35700786](https://pubmed.ncbi.nlm.nih.gov/35700786/).
58. Liang F, Pan Y, Peng H, Zeng M, Huang C. Time-Space Simulation, Health Risk Warning and Policy Recommendations of Environmental Capacity for Heavy Metals in the Pearl River Basin, China. Int J Environ Res Public Health. 2022;19(8). doi: 10.3390/ijerph19084694. PMID: [35457560](https://pubmed.ncbi.nlm.nih.gov/35457560/).
