# Supplementary material for: Assessment of heavy metal pollution in sediments from the urban section of Yihe River, Linyi City, China
Source: PLoS One. 2025 Feb 13;20(2):e0318579. doi: 10.1371/journal.pone.0318579 (PMC11824964; doi:10.1371/journal.pone.0318579)
Supplement: Attachment — Submitted filename: PLOS 1 REVEIW 24.docx [file pone.0318579.s007.docx]

Title: Heavy metal pollution in sediments from the urban section of Yihe River, Linyi City, Shandong, China

The team should adopt a chemist to execute risk assessment, adding value to the publication.

Abstract

Measurement of potentially toxic substances i.e. heavy, metals levels in sediment differs from what is in the water levels. Comparisons are crucial for discussion towards health

**Introduction**

Since this is an ecological risk assessment, the three phases:

- Phase 1 - Problem Formulation Information was documented.
- Phase 2 - Analysis was also well documented
- Phase 3 - Risk Characterization: this was not well discussed and the target populations should be clearly articulated. Use Hazard index and reference doses for vulnerable populations.

The above will enrich the introduction, methods and discussion.

Enrich discussions by comparing sediment samples with airborne and water samples and why sediment are crucial source.

**Methods**

These are detailed and well-written.

Limitations to methods and samples are missing. Sediment samples are usually not representative of water sample amounts of pollutants even though Toxicology assessments document risks for exposure through water uptake.

**Results**

The distribution of HMs in surface sediments should be presented in tables and possibly also show the max allowable limits by various associations.

The spatial maps show individual pollutant distribution so that the heavily polluted sections of the river are displayed, linked to sources of contamination and strategies of how to control can be displayed.

Risk assessment can be displayed using probability index, global rand scatter plots and Local Moran's I clustering plots

**Discussions**

You discussed sources, distribution and unfortunately the health burden was not clearly articulated.

**Conclusions**

Information on health burden not presented.
