# Supplementary material for: Assessment of heavy metal pollution in sediments from the urban section of Yihe River, Linyi City, China
Source: PLoS One. 2025 Feb 13;20(2):e0318579. doi: 10.1371/journal.pone.0318579 (PMC11824964; doi:10.1371/journal.pone.0318579)
Supplement: Attachment — Submitted filename: Response to Reviewers.docx [file pone.0318579.s009.docx]

**Response to Reviewers**

Dear Editor and Reviewers:

Thank you for your letter and for the reviewers' comments concerning our manuscript entitled “**Assessment and source analysis of heavy metal pollution in sediments from the urban section of Yihe River, Linyi City, Shandong, China**” (**Manuscript Number: PONE-D-24-25973**). Those comments are all valuable and very helpful for revising and improving our paper, as well as the important guiding significance to our researches. We have studied all comments carefully and have made correction that we hope meet with approval. The revised sections have been distinctly highlighted in red within the paper. The main corrections in the paper and the responds to the reviewers' comments are as following:

**Responds to the reviewer' s comments:**

**Reviewer 1:**

**Question 1. Scientific value of research topics**

Although the manuscript focuses on the risk assessment of heavy metal pollution in the Linyi section of the Yihe River, which is of great significance to environmental protection and water resources management, the scope of the study is relatively limited, focusing only on heavy metal pollution in sediments, without considering the migration and transformation of heavy metals in water and their impact on aquatic ecosystems. In addition, the assessment and risk assessment of heavy metal pollution usually require long-term monitoring and comprehensive analysis, while the short-term and single-point sampling in this study may not be enough to fully reflect the spatial and temporal distribution characteristics of heavy metal pollution. Therefore, the scientific value of research topics is limited to a certain extent.

**Response:** Thank you very much for your valuable comments. This study mainly focuses on the risk assessment and source analysis of heavy metal pollution in the Linyi section of the Yihe River. Although the scope is limited, it is of great significance for regional environmental protection and water resource management. In addition, our current data is limited, but we will continue to monitor and analyze heavy metal pollution in the area in the future.

**Question 2.** **Innovation of research methods**

In terms of research methods, although pollution load index, geological accumulation index and other methods are used to evaluate heavy metal pollution, these methods are relatively traditional and do not show significant innovation in research methods. Especially in the part of source apportionment, the methods of correlation analysis and absolute principal components-multiple linear regression (APCs-MLR) can identify the sources of heavy metals, but these methods have been widely used in the field of environmental science, which do not reflect the uniqueness and innovation of research methods.

**Response:** Thank you very much for your comments. Although our research methods are relatively traditional and widely used, they have also been proven to be effective for heavy metal pollution assessment and source apportionment. Of course, we will strive to develop some new models and evaluation methods in the future.

**Question 3.** **The practicability of the research results**

Although the research results show that there is no heavy metal pollution in most areas of the main stream of yi river, and the ecological risk is low, the ecological risk of tributaries and central islands is high. However, these research results have limited practical guiding significance for local environmental management departments and relevant state departments. On the one hand, the research results did not put forward specific pollution control and treatment measures; On the other hand, the research results do not provide effective policy suggestions for the control and governance of pollution sources. Therefore, the research results are not practical enough to be directly applied to environmental management and policy formulation.

**Response:** Thank you very much for your comments. We have deeply understood and attached great importance to the shortcomings of the research you pointed out in proposing specific pollution control measures and policy recommendations. In response to your feedback, we have made corresponding supplements and improvements in the revised manuscript. For example, strengthening the supervision and performance evaluation of the mechanism for phasing out outdated production capacity, enhancing environmental governance supervision of key polluting industries, formulating and implementing strict emission management policies, reducing heavy metal emissions from motor vehicle exhaust, prohibiting the use of fertilizers with excessive heavy metals, and so on. These contents are in the last paragraph of the discussion section and have been highlighted in red font.

**Reviewer 2:**

**Question 1. Abstract**

Measurement of potentially toxic substances i.e. heavy, metals levels in sediment differs from what is in the water levels. Comparisons are crucial for discussion towards health.

**Response:** Thank you for your feedback. We have considered your suggestions and supplemented the significance of studying heavy metals in sediments and added a human health risk assessment model to assess the health risks of heavy metals in sediments for local residents. The revisions are in the first paragraph of the Abstract section.

**Question 2. Introduction**

Since this is an ecological risk assessment, the three phases:

- Phase 1 - Problem Formulation Information was documented.
- Phase 2 - Analysis was also well documented
- Phase 3 - Risk Characterization: this was not well discussed and the target populations should be clearly articulated. Use Hazard index and reference doses for vulnerable populations.

The above will enrich the introduction, methods and discussion.

Enrich discussions by comparing sediment samples with airborne and water samples and why sediment are crucial source.

**Response:** Thank you for your recognition and affirmation of our work. We have considered your suggestion and added the health risks of heavy metals to the target population (local residents) and discussed the vulnerable populations (children). We also added the reasons why sediment is a crucial source. The revisions are in the first paragraph of the Introduction section.

**Question 3.Methods**

These are detailed and well-written.

Limitations to methods and samples are missing. Sediment samples are usually not representative of water sample amounts of pollutants even though Toxicology assessments document risks for exposure through water uptake.

**Response:** Thank you very much for your careful review of our study and your valuable comments. We acknowledge that there is indeed a complex relationship between the pollutant content in sediments and the content in water bodies, and they cannot be simply equated or represented. Sediments are both "sinks" and "sources" of pollutants, and their pollutant concentrations may be affected by many factors. However, we are sorry that in this study, we do not have data on heavy metals in water bodies, so we cannot add them to the manuscript. We added the explanation in the fourth paragraph of the Sample collection and analysis section of the Materials and Methods chapter of the manuscript that no water sample data were collected.

**Question 4. Results**

The distribution of HMs in surface sediments should be presented in tables and possibly also show the max allowable limits by various associations.

The spatial maps show individual pollutant distribution so that the heavily polluted sections of the river are displayed, linked to sources of contamination and strategies of how to control can be displayed.

Risk assessment can be displayed using probability index, global rand scatter plots and Local Moran's I clustering plots.

**Response:** Thank you for your valuable feedback. We have taken your suggestion into consideration and made the following changes to the manuscript. We included the heavy metal concentrations at each sampling point (Table S2) in the supplementary material. We have updated other association max allowable limits (Table 3). And we incorporate probability distribution into risk assessment (Fig 3b) and supplement Moran’s cluster diagrams for ecological and toxic risks (Fig 6). Revisions have been highlighted in red in the manuscript.

**Question 5. Discussions**

You discussed sources, distribution and unfortunately the health burden was not clearly articulated.

**Response:** Thank you very much for your thorough review and valuable comments. We have fully considered your suggestion and added the discussion on health risks to the fourth paragraph of the Discussion section.

**Question 6. Conclusion**

Information on health burden not presented.

**Response:** Thank you for your valuable feedback. We have taken your suggestion into consideration and added the results on health burden in the fourth paragraph of the Conclusion section.

We have carefully reviewed the comments provided by the reviewers and made the necessary modifications and explanations. We have diligently worked to enhance the manuscript by implementing specific changes. These alterations do not affect the content or structure of the paper.

We sincerely appreciate the diligent efforts of Editors and Reviewers, and we hope that the revision will meet with approval.

Once again, thank you very much for your comments and suggestions.

Yours sincerely,

Liping Zhang
