# Supplementary material for: Assessment of heavy metal pollution in sediments from the urban section of Yihe River, Linyi City, China
Source: PLoS One. 2025 Feb 13;20(2):e0318579. doi: 10.1371/journal.pone.0318579 (PMC11824964; doi:10.1371/journal.pone.0318579)
Supplement: Attachment — Submitted filename: Response_to_Reviewers_auresp_2.docx [file pone.0318579.s011.docx]

**Response to Reviewers**

Dear Editor and Reviewers:

Thank you for your letter and for the reviewers' comments concerning our manuscript entitled “**Assessment and source analysis of heavy metal pollution in sediments from the urban section of Yihe River, Linyi City, Shandong, China**” (**Manuscript Number: PONE-D-24-25973R1**). Those comments are all valuable and very helpful for revising and improving our paper, as well as the important guiding significance to our researches. We have studied all comments carefully and have made correction that we hope meet with approval. The revised sections have been distinctly highlighted in red within the paper. The main corrections in the paper and the responds to the reviewers' comments are as following:

**Responds to the editor' s comments:**

**Question 1.** The Discussion section in its current form is too regionally focused. Most of the studies cited are exclusively from China, which limits the broader applicability and significance of your findings. Please compare your results with studies from other parts of the world to strengthen this section.

**Response:** Thank you very much for your valuable comments. We have added studies from world regions for comparison in the manuscript, and we firmly believe that this will make the findings more broadly applicable and meaningful.

**Question 2.** Compare your results of heavy metals in sediments with benchmarks such as the average shale values, toxicity reference values, and consensus-based sediment quality guidelines. Using the “China Soil Standards – Farmland” is not appropriate in this context since these are sediments from a river system.

**Response:** Thank you for your valuable comments. We have deleted the part about “China Soil Standards - Farmland” in the manuscript and added the average shale value as the evaluation standard. Since the toxicity reference values are shown in the Risk Assessment Methods section of the manuscript, the toxicity reference valuesare not added here.

**Question 3.** PCA results could be better presented graphically, for example, using biplots, as this is a best practice for clarity and accessibility. If necessary, the detailed results currently shown in Table 8 can be moved to the supplementary files. Graphical presentation makes PCA loadings much easier to interpret.

**Response:** Thank you very much for your comments. We have added the PCA biplots to the manuscript and moved the table with eigenvalues and contributions to the supplementary files.

**Question 4.** Hierarchical Cluster Analysis (HCA) could provide additional insights into the data. Consider incorporating it for a more comprehensive analysis.

**Response:** Thank you very much for your valuable comments. We have carefully considered your suggestion and combined it with the HACA Dendrogram (Fig 7) for data analysis.

**Responds to the reviewer' s comments:**

**Reviewer 1:**

**Question 1.** Design Logic. Given that the authors state, "Due to research design and resource constraints, this study did not collect water samples for testing. Therefore, we cannot directly analyze the relationships and differences between heavy metals in water and sediments. However, through detailed sediment analysis, we still revealed some important characteristics of heavy metal contamination in the area." From the perspective of scientific rigor, the authors' research design is inherently flawed, with subsequent conclusions primarily derived from inference rather than actual experimental data.

**Response:** Thank you for your comments. We admit that there may be a logical problem. Due to funding reasons, we did not collect water samples in the early design. We read a lot of literature, summarized and sorted out the experience of predecessors, and combined with the actual situation of Yihe and Linyi City, then we conducted a reasonable analysis.

**Question 2.** Data Quality. The authors emphasize quality control in data analysis but neglect repetitive testing of samples. As a result, the statistical stability and variability of the data cannot be confirmed.

**Response:** Thank you for your comments. We apologize for not making it clear in the manuscript. When collecting samples, we collected three samples from the area around each sampling point and mixed them evenly before measuring the obtained sampling data. We have added these details to the manuscript.

**Question 3.** The graphical materials provided by the authors are of extremely low resolution, making it impossible to clearly understand the data acquisition information.

**Response:** Thank you for your valuable comments. This is not intentional on our part, but because before uploading the images, they need to be processed by the image processing website designated by the journal, which may reduce the image resolution.

**Reviewer 3:**

**Question 1.** Remove all instances of HEAVY metals and alter to metals and metalloids, according to IUPAC standards.

**Response:** Thank you for your valuable comments. We have changed all "heavy metals " in the manuscript to "metals or metalloids ".

**Question 2.** Abstract; "The HMs were evaluated by measuring the pollution load index, geoaccumulation index, etc. " etc is not adequate, rewrite. Overall comment: Do not begin sentences with abbreviations, such as HM, alter.

**Response:** Thank you very much for your comments. We rewrote the methods in the Abstract section and changed all abbreviations in the manuscript to full names.

**Question 3.** Correct: MATERIAL and methods, no S

**Response:** Thank you very much for your comments. We have corrected this part.

**Question 4.** How were the sediments sampled, at what depth?

**Response:** Thanks for your comments. The Sample collection and analysis section of the manuscript has already described how to collect samples and the sampling depth.

**Question 5.** Correct "detection limits" to "limits of detection".

**Response:** Thank you very much for your valuable comments. We have corrected this in the manuscript.

**Question 6.** Alter units to IUPAC standards, not mg/kg, but instead, mg kg-1.

**Response:** Thank you very much for your comments. We have corrected these contents in the manuscript and marked them in red font.

**Question 7.** Remove the Human Health Risk assessment, humans do not ingest this sediment. Or at least, maintain only dermal contact.

**Response:** Thank you for your valuable feedback. We have taken your suggestion into consideration and deleted the content about human health risk assessment.

**Question 8.** Indicate how data normality was checked in order to employ Pearson correlation.

**Response:** Thank you for your valuable comments. We are sorry that we overlooked the conditions for using person correlation analysis before using it. After verification, the data did not conform to the normal distribution well, so person correlation analysis could not be used. We changed it to spearman correlation analysis in the manuscript. Thank you again for your suggestions, which helped us avoid the mistake.

**Responds to the manuscript' s comments:**

**Question 1.** Consider revising this title as suggested. It is too long and redundant.

**Response:** Thank you for your valuable feedback. We have taken your suggestion into consideration and shortened the title.

**Question 2.** Indicate the analytical technique used for the measurement.

**Response:** Thank you for your valuable comments. We have added the analytical technique used for the measurement in the Abstract section.

**Question 3.** Please calculate the enrichment factor (EF) as well. There are already of studies from China, and you should be able to have the Reference metals with established concentrations.

**Response:** Thank you for your valuable comments. We are sorry that this method cannot be used because the actual values of the reference elements required for the enrichment factor method were not determined during the preliminary design and measurement process.

**Question 4.** Which software was used for this analysis? Please indicate the version, developer of the software and their location.

**Response:** Thank you for your valuable comments. We have added information about the software, version and location used in the analysis in the manuscript.

**Question 5.** About some grammatical errors or minor modifications.

**Response:** Thank you for your valuable comments. We have corrected these contents in the manuscript.

We have carefully reviewed the comments provided by the reviewers and made the necessary modifications and explanations. We have diligently worked to enhance the manuscript by implementing specific changes. These alterations do not affect the content or structure of the paper.

We sincerely appreciate the diligent efforts of Editors and Reviewers, and we hope that the revision will meet with approval.

Once again, thank you very much for your comments and suggestions.

Yours sincerely,

Liping Zhang
