# Supplementary material for: Assessment of heavy metal pollution in sediments from the urban section of Yihe River, Linyi City, China
Source: PLoS One. 2025 Feb 13;20(2):e0318579. doi: 10.1371/journal.pone.0318579 (PMC11824964; doi:10.1371/journal.pone.0318579)
Supplement: Attachment — Submitted filename: Response_to_Reviewers_auresp_3.docx [file pone.0318579.s012.docx]

**Response to Reviewers**

Dear Editor:

Thank you for your letter and for the reviewers' comments concerning our manuscript entitled “**Assessment of heavy metal pollution in sediments from the urban section of Yihe River, Linyi City, China**” (**Manuscript Number: PONE-D-24-25973R2**). Those comments are all valuable and very helpful for revising and improving our paper, as well as the important guiding significance to our researches. We have studied all comments carefully and have made correction that we hope meet with approval. The revised sections have been distinctly highlighted in red within the paper. The main corrections in the paper and the responds to the reviewers' comments are as following:

**Responds to the Editor' s comments:**

**Question 1.** **Clarification on Normality Testing.**

The reviewer comment, “Indicate how data normality was checked in order to employ Pearson correlation,” was not sufficiently addressed. Please clearly specify in the manuscript which normality test was used (e.g., Shapiro-Wilk, Kolmogorov-Smirnov). Use of an inappropriate normality test can lead to false assumptions about the data distribution and, subsequently, the application of incorrect statistical methods.

**Response:** Thank you very much for your valuable comments. Since the data did not follow a normal distribution, we changed the Pearson correlation analysis to a Spearman correlation analysis. And we have added this content in the STATISTICAL ANALYSIS section, which you can find on page 11 of the manuscript.

**Question 2.** **Steps for Spearman Correlation Analysis.**

Spearman correlation is a non-parametric method that evaluates monotonic relationships by ranking the data. Please indicate the exact steps undertaken to implement it in your analysis. For instance, note any rank transformations or adjustments for tied ranks, as these are required for this method.

**Response:** Thank you for your valuable comments. We have added detailed steps on Spearman correlation coefficient in the STATISTICAL ANALYSIS section on page 11 of the manuscript.

**Question 3.** **Expanded Explanation for HACA Results.**

Please elaborate further on the HACA (Hierarchical Cluster Analysis) results on page 24, sentence after Table 6. Specifically:

(a) Primary Clusters (broad groups): Discuss how similar sources (e.g., Cu and Hg from industrial activities) or comparable environmental behavior and mobilization processes (e.g., Pb, Ni, Zn, and Cr adsorbing onto organic matter or clay minerals) could explain these groupings.

(b) Sub-Clusters (Finer Groupings): Explain how these may reflect either geochemical similarities (e.g., Cr and Ni) or distinct combinations of anthropogenic and natural contributions (e.g., Cd and Pb arising from industrial emissions with varying inputs from transportation).

(c) Please revise the STATISTICAL ANALYSIS section to indicate that HACA was performed.

**Response:** Thank you very much for your comments. We have supplemented the information about HACA in the DISCUSSION section, described the primary clusters and sub-clusters, and discussed possible sources. In the STATISTICAL ANALYSIS section, we indicated that this study used HACA.

We have carefully reviewed the comments provided by the reviewers and made the necessary modifications and explanations. We have diligently worked to enhance the manuscript by implementing specific changes. These alterations do not affect the content or structure of the paper.

We sincerely appreciate the diligent efforts of Editors and Reviewers, and we hope that the revision will meet with approval.

Once again, thank you very much for your comments and suggestions.

Yours sincerely,

Liping Zhang
